# Metabolic engineering of proanthocyanidin production by repressing the isoflavone pathways and redirecting anthocyanidin precursor flux in legume

**DOI:** 10.1111/pbi.12524

**Published:** 2016-01-24

**Authors:** Penghui Li, Qiang Dong, Shujun Ge, Xianzhi He, Jerome Verdier, Dongqin Li, Jian Zhao

**Affiliations:** ^1^ National Key Laboratory of Crop Genetic ImprovementHuazhong Agricultural UniversityWuhanChina; ^2^ College of AgronomyAgricultural University of HebeiBaodingChina; ^3^ Plant Biology DivisionThe Samuel Roberts Noble FoundationArdmoreOKUSA; ^4^ Department of Plant and Microbial BiologyNorth Carolina State UniversityRaleighNCUSA; ^5^ Shanghai Center for Plant Stress Biology, Shanghai Institutes for Biological SciencesChinese Academy of ScienceShanghaiChina

**Keywords:** legume, proanthocyanidin, isoflavone, transcription factor, metabolic engineering

## Abstract

*MtPAR* is a proanthocyanidin (PA) biosynthesis regulator; the mechanism underlying its promotion of PA biosynthesis is not fully understood. Here, we showed that MtPAR promotes PA production by a direct repression of biosynthesis of isoflavones, the major flavonoids in legume, and by redirecting immediate precursors, such as anthocyanidins, flux into PA pathway. Ectopic expression of *MtPAR* repressed isoflavonoid production by directly binding and suppressing isoflavone biosynthetic genes such as *isoflavone synthase* (*IFS*). Meanwhile, MtPAR up‐regulated PA‐specific genes and decreased the anthocyanin levels without altering the expression of anthocyanin biosynthetic genes. MtPAR may shift the anthocyanidin precursor flux from anthocyanin pathway to PA biosynthesis. MtPAR complemented PA‐deficient phenotype of Arabidopsis *tt2* mutant seeds, demonstrating their similar action on PA production. We showed the direct interactions between MtPAR, MtTT8 and MtWD40‐1 proteins from *Medicago truncatula* and *Glycine max*, to form a ternary complex to *trans*‐activate PA‐specific *ANR* gene. Finally, *MtPAR* expression in alfalfa (*Medicago sativa*) hairy roots and whole plants only promoted the production of small amount of PAs, which was significantly enhanced by co‐expression of *MtPAR* and *MtLAP1*. Transcriptomic and metabolite profiling showed an additive effect between *MtPAR* and *MtLAP1* on the production of PAs, supporting that efficient PA production requires more anthocyanidin precursors. This study provides new insights into the role and mechanism of MtPAR in partitioning precursors from isoflavone and anthocyanin pathways into PA pathways for a specific promotion of PA production. Based on this, a strategy by combining MtPAR and MtLAP1 co‐expression to effectively improve metabolic engineering performance of PA production in legume forage was developed.

**Highlights**

- Proanthocyanidin (PA) production in alfalfa was achieved by manipulating two MYB transcription factors.
- MtPAR directly binds *isoflavone synthase* (*IFS*) promoter and down‐regulates *IFS* and isoflavone biosynthesis.
- *MtPAR* specifically up‐regulates PA biosynthesis without affecting anthocyanin‐specific genes.
- MtPAR and MtLAP1 collaborate in reprocessing isoflavone pathway and enhancing anthocyanidin precursors flux into PA pathway to promote PA production for breeding pasture boat‐safe alfalfa.

## Introduction

Plants produce abundant flavonoids, such as anthocyanins and proanthocyanidins (PAs; also called condensed tannins), in seed coats, leaves, fruits, flowers and barks (Ariga *et al*., 1981; Dixon *et al*., 2005; Gabetta *et al*., 2000; Gu *et al*., 2004). These flavonoids play protective roles in battles against microbial pathogen and pest attacks, UV protection and attracting insect pollinators (Dixon *et al*., 2005; Santos‐Buelga and Scalbert, 2000; Zhao *et al*., 2005). The modest levels of PAs in the leaves and stems of protein‐rich forage crops, such as alfalfa (*Medicago sativa*), can prevent lethal pasture bloat of ruminant animals, enhance ruminant nutrition and reduce protein degradation in silage and thus is an important agronomic trait (Coblentz and Grabber, 2013; Lees, 1992; Zhao and Dixon, 2009). Lethal pasture bloat of legume forage alone causes more than hundred million dollars of global loss annually, mostly due to livestock grazing on alfalfa grasslands (Coblentz and Grabber, 2013). Engineering PA production in alfalfa shoots has been a research target for long time, and extensive efforts have been made to engineer PA production in alfalfa (*Medicago sativa*) for animal diet improvement (Lees, 1992; Dixon *et al*., 2005; Escaray *et al*., 2014; Yuan and Grotewold, 2015). While anthocyanin production has been engineered successfully (Butelli *et al*., 2008; Peel *et al*., 2009), attempts for PA production have so far only led to enhanced anthocyanin or less than expected level of PA accumulation (Verdier *et al*., 2012). The limited knowledge about the complex biosynthesis and regulation of PA biosynthesis is regarded as a major obstacle (Dixon *et al*., 2005; Zhao *et al*., 2010).

**Figure 1:**
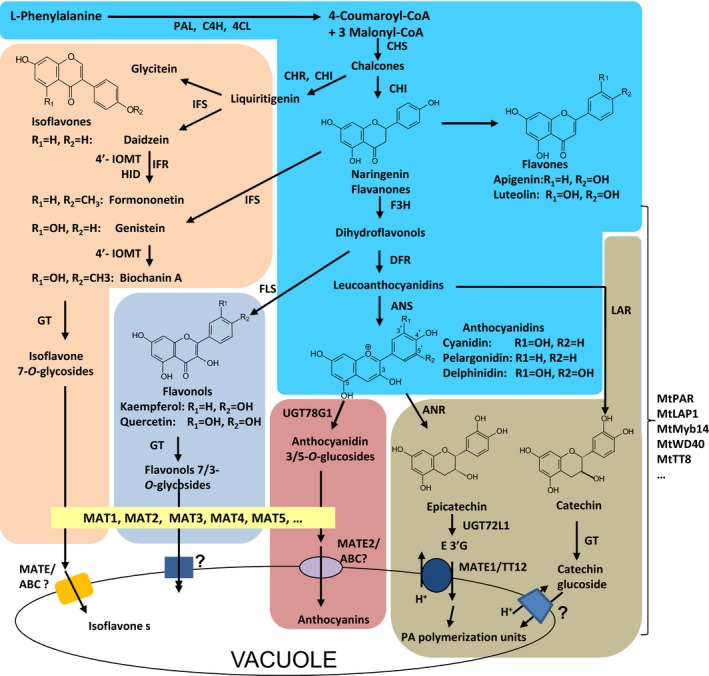
Scheme of (iso)flavonoid/proanthocyanidin biosynthesis in *M. truncatula*. PAL, phenylalanine ammonia‐lyase; C4H, cinnamate 4‐hydroxylase; 4CL, 4‐coumarate CoA ligase; CHS, chalcone synthase; CHI, chalcone isomerase; CHR, chalcone reductase; IFS, isoflavone synthase; IOMT, isoflavonoid *O*‐methyltransferase; HID, 2‐hydroxyisoflavanone dehydratase; F3H, flavanone 3‐hydroxylase; FLS, flavonol synthase; DFR, dihydroflavonol 4‐reductase; ANS, anthocyanidin synthase; ANR, anthocyanidin reductase; OMT,* o*‐methyltransferase; UGT, UGT, UDP‐glucose: flavonoid glycosyltransferase; MAT, malonyl‐CoA: flavonoid glucoside acyltransferase; MATE1/TT12, multidrug and toxic extrusion protein1/transparent testa 12.

PAs with their monomeric building blocks, catechins and epicatechins and anthocyanins are derived from the common flavonoid biosynthetic pathway and share the same precursors leucoanthocyanidins, which either are reduced to give catechin by leucoanthocyanidin reductase (LAR), or are oxidized to generate anthocyanidins (such as cyanidin, pelargonidin and delphinidin) by anthocyanidin synthase (ANS) (Figure 1). It has been suggested that competition between the PA and anthocyanin biosynthesis pathways from anthocyanidins could be the key mechanism for PA biosynthesis (Lepiniec *et al*., 2006; Verdier *et al*., 2012). Anthocyanidins are either immediately modified by glycosylation to give anthocyanins by anthocyanidin 3‐*O*‐glycosyltransferases (UGTs) or reduced to generate flavan‐3‐ols (such as epicatechin) by anthocyanidin reductase (ANR) for PA biosynthesis (Xie *et al*., 2003) (Figure 1). In supporting such a hypothesis, Arabidopsis *ANR* mutant (*ban*) seeds accumulate more anthocyanins than wild‐type seeds, while *ugt78g1* mutant seeds accumulate more PAs (Lee *et al*., 2005). The inverse relationship between the expression of *ANR* and *UGT78G1* has been proposed to switch anthocyanidins into either PA or anthocyanin biosynthetic pathways (Lee *et al*., 2005). Many major anthocyanin and PA synthetic genes, such as *ANS*,* LAR*,* ANR* and *UGT78G1,* are tightly regulated at transcription level (Xu *et al*., 2013, 2014). One of the major regulatory mechanisms is the formation of ternary complexes including MYB, basic helix–loop–helix (bHLH) and WD40‐repeat transcription factors (MBW complex) (Walker *et al*., 1999; Terrier *et al*., 2009; Albert *et al*., 2014, 2015; Hancock *et al*., 2014; Lepiniec *et al*., 2006; Baudry *et al*., 2004). Among them, R2R3 MYBs, such as TRANSPARENT TESTA 2(TT2), Medicago *PROANTHOCYANIDIN REGULATOR* (MtPAR) and LEGUME ANTHOCYANIN PRODUCTION 1(MtLAP1), may determine the specificity of the complexes (Albert *et al*., 2014). Furthermore, as major flavonoids in legume plants, isoflavone biosynthesis also shares common precursors, such as chalcones and naringenin flavanones with the anthocyanin pathway. Therefore, isoflavone biosynthesis competes with anthocyanidin biosynthesis (Shelton *et al*., 2012) (Figure 1). However, the mechanisms underlying the regulation of isoflavone biosynthesis and its competition with anthocyanin pathway are largely unknown (Shelton *et al*., 2012) (Figure 1). How such a competition for precursor pools generated from the common phenylpropanoid pathway cooperate with the complex regulation of anthocyanin, isoflavone and PA biosynthesis remains elusive.

In a model legume *Medicago truncatula* and forage alfalfa, constitutive expression of *MtLAP1* strongly induces *UGT78G1* and two anthocyanin acyltransferase genes *MaT5* and *MaT6*, resulting in a massive production of anthocyanidins and anthocyanins but no PAs detected (Benedito *et al*., 2008; Peel *et al*., 2009; Zhao *et al*., 2011). Expression of *MtPAR* induced a significant level of PAs in *M. truncatula* hairy roots and resulted in detectable level of PAs in transgenic alfalfa plants, although the PA levels in alfalfa shoots remain too low to have a pasture bloat protection function (Verdier *et al*., 2012). *MtLAP1* was characterized as an important regulator of anthocyanin biosynthesis (Peel *et al*., 2009), but MtPAR is regarded as a key regulator of PA biosynthesis (Verdier *et al*., 2012). As a seed‐specifically expressed MYB factor, MtPAR positively regulates flavonoid‐PA pathway genes via a probable activation of MtWD40‐1, an ortholog of Arabidopsis TTG1 that plays an indispensible role in regulating PA production in *M. truncatula* (Walker *et al*., 1999; Pang *et al*., 2009; Verdier *et al*., 2012). However, the detailed mechanisms by which MtPAR promoted PA production are not well understood (Verdier *et al*., 2012). The level of PAs in MtPAR transgenic alfalfa was very low, far lower than 20 mg of PAs/g dry weight biomass, which is estimated as the minimal levels to provide effective bloat protection (Verdier *et al*., 2012). In this study, we tried to reveal the mechanisms underlying MtPAR regulation of PA biosynthesis and its application in metabolic engineering of PAs in alfalfa in a new strategy. We showed that MtPAR partitions precursor pools between isoflavonoid and flavonoid, anthocyanin and PA pathways in different legume species, Medicago, alfalfa and soybean (*Glycine max*). We analysed the regulatory complex formed by interaction of MtPAR with MtTT8 and MtWD40‐1 leading to activation of PA pathway genes. Finally, by co‐expression of *MtLAP1* and *MtPAR* in alfalfa, we observed an additive effect of co‐expression of *MtLAP1* and *MtPAR* in alfalfa on PA production by increasing the pool of precursors shared between anthocyanin and PA biosynthetic pathways. Using the strategy, we were able to increase the PA production to about 2 mg PAs/g dry weight mass.

## Results

### Ectopic expression of MtPAR affected expression of genes involved in the PA pathway

**Figure 2:**
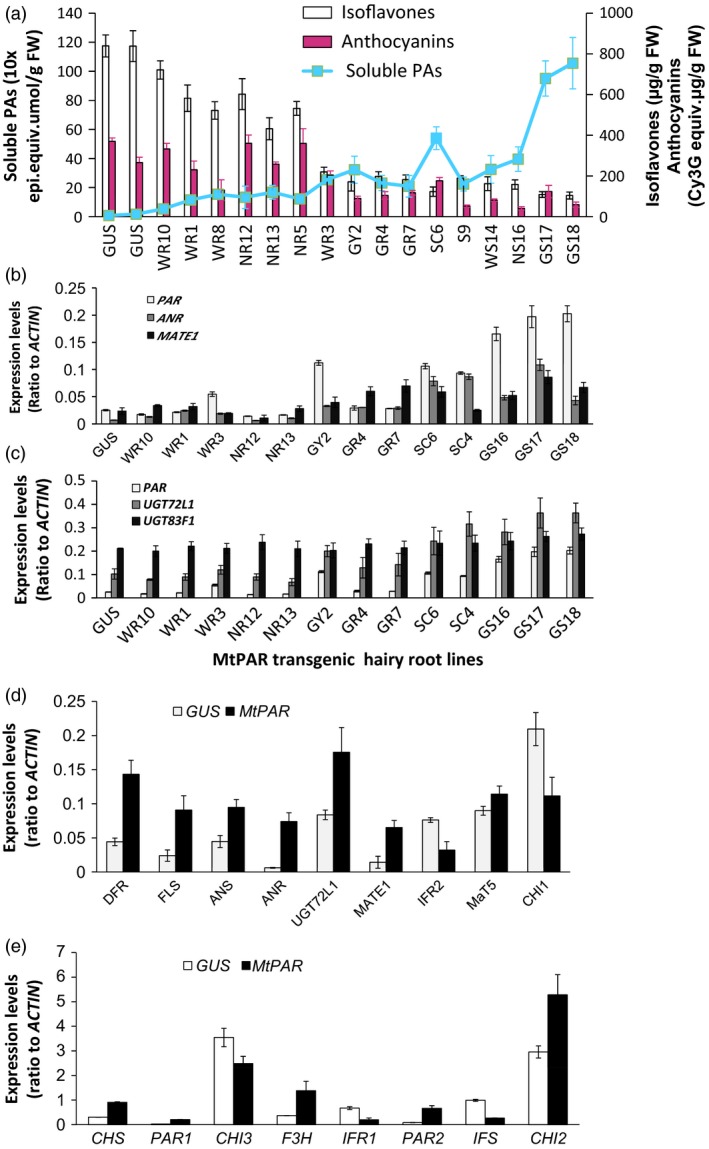
PA, anthocyanin and isoflavone production in Medicago hairy roots over‐expressing *MtPAR*. (a) Accumulation of soluble PAs, anthocyanins and isoflavones in *MtPAR* transgenic hairy roots as compared with GUS control. Data represent mean ± SD of at least three biological replicates. (b) Expression of *MtPAR* and two PA‐specific genes *MtANR* and *MtMATE1* in *MtPAR* hairy root lines. (c) Expression of PA‐specific glycosyltransferase gene *UGT72L1* and anthocyanin‐specific glycosyltransferase gene *UGT83F1* in *MtPAR* hairy root lines. (d, e) Expression of PA and isoflavone biosynthesis‐related gene in *MtPAR* transgenic hairy roots as compared with GUS control. Abbreviations for genes are the same as in Figure 1. PAR1 and PAR2 represent MtPAR gene expression using two pairs of primers in qRT‐PCR. *CHI1* and *CHI2*, and *IFR1* and *IFR*2 represent, respectively, two major chalcone isomerases and two isoflavone synthases. Data are expressed as mean ± SD from at least three independent experiments.

More than 24 independent *M. truncatula* transgenic hairy root lines ectopically expressing *MtPAR* were generated and analysed for their content of soluble PAs, anthocyanins and isoflavones. In the control lines, over‐expressing the *GUS* gene, no PA was detected, while high amount of isoflavones was observed. On the contrary, in the lines ectopically expressing *MtPAR*, we observed an increase of the soluble PA contents and decreased amounts of isoflavones compared to GUS controls. Overall, we observed a negative correlation between the accumulation of soluble PAs and isoflavones (correlation coefficiency *r* ^2^ = 0.75) (Figure 2a). The level of anthocyanins in these transgenic lines was also clearly affected by the increase of soluble PAs (Figure 2a). We observed about one‐third hairy root lines showed high *MtPAR*; meanwhile, their anthocyanin levels were much lower, resulting in hairy root with a green colour. Around one‐third of transgenic hairy root lines displayed red colour with large amounts of anthocyanins, and they also had lower PA production. The remaining transgenic lines displayed moderate accumulation of PAs, with a red‐green hairy root colour phenotype (Figures S1 and S2). Transcript and metabolite profiling of transgenic lines showed a positive correlation between PA production and expressions of *MtPAR*; greener hairy roots usually had higher PA contents, whereas reddish hairy roots had fewer PA levels. Therefore, apparently these hairy root lines with more PAs were associated with reduced anthocyanin accumulation in MtPAR hairy roots; there exists a negative correlation (*r* ^2^ = 0.68). This correlation was also observed between PA accumulation and other PA related genes, such as genes involved in biosynthesis (*ANR*), modification (*UGT72L1*) and transport (*MtMATE1*) (Figure 2b,c).

We examined the transcriptional levels of anthocyanin related genes such as modification enzymes UGTs (*MtUGT78G1*, probe set Mtr.39747.1.S1_at), acyltransferases (*MaT5,* probe set Mtr.19945.1.S1_at and *MaT6,* probe sets Mtr.44986.1.S1_at and Mtr.37182.1.S1_at) and transporter (*multidrug and toxin extrusion 2, MATE2,* probe set Mtr.51063.1.S1_at). Most of these genes did not display obvious changes in expression in hairy root lines over‐expressing *MtPAR* according to microarray and quantitative reverse transcription–polymerase chain reaction (qRT–PCR) results (Table S1), some of them even showed decreased expression in hairy root lines with higher *MtPAR* expression and PA production. Therefore, apparently a negative correlation also exist between PA and anthocyanin accumulation in *MtPAR* hairy root lines.

To understand the impact of *MtPAR* ectopic expression on the whole flavonoid pathway, we compared expression profiles of genes involved in the flavonoid pathway between *MtPAR*‐expression lines with high PA content with *GUS* control hairy root lines (Figure 2d,e). We observed that genes involved in the early flavonoid pathway to generate common precursors of PA, anthocyanin and isoflavone, were up‐regulated when *MtPAR* was ectopically expressed such as *dihydroflavonol 4‐reductase (DFR*), *chalcone isomerase* (*CHI2*) and *flavanone 3‐hydroxylase* (*F3H*). On the contrary, we noticed that most of the genes involved in the isoflavone biosynthesis were down‐regulated such as *isoflavone reductase 1* and *2* (*IFR1 and IFR2)* and *isoflavone synthase* (*IFS*). This down‐regulation of genes involved in the isoflavone biosynthesis in *MtPAR‐lines* explained the negative correlation between PA and isoflavone contents.

### MtPAR repressed the isoflavone production in Medicago hairy roots

**Figure 3:**
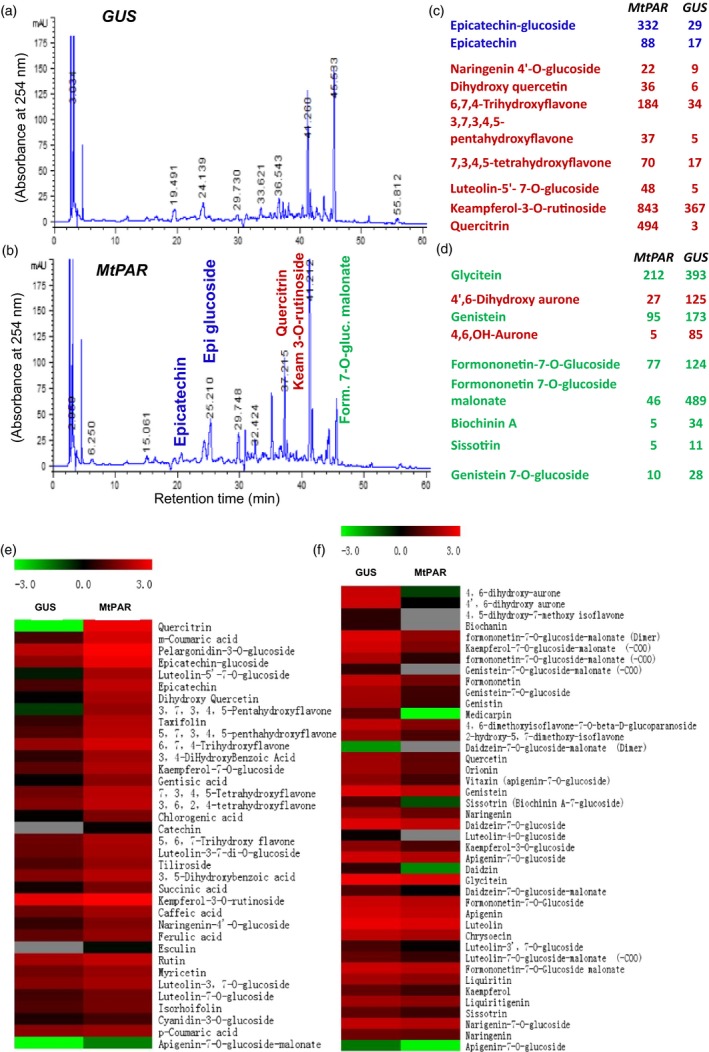
Flavonoid profiles in transgenic *M. truncatula* hairy roots over‐expressing *MtPAR*. (a, b) HPLC chromatographs of flavonoids in *MtPAR* transgenic hairy roots and GUS control. Major flavonoids such as formononetin 7‐*O*‐glucoside malonate, formononetin 7‐*O*‐glucoside, glycitein, 4′,6‐dihydroxy aurone, genistein and epicatechin 3′‐*O*‐glucoside and epicatechin, kaempferol 3‐*O*‐rutinoside, quercitrin are indicated in HPLC traces. (c, d) Flavonoid and isoflavonoid contents in hairy roots over‐expressing *MtPAR* and *GUS*. Values represent mean ± SD from three biological replicates. (e, f) Heat map analysis of flavonoids in hairy roots over‐expressing *MtPAR* and *GUS*. Data are expressed as mean ± SD from at least three independent experiments.

We then investigated details regarding how *MtPAR* expression negatively impacts isoflavone accumulation. Medicago hairy roots also produced large amounts of isoflavonoids, similar to these identified from suspension cell cultures derived from their roots (Farag *et al*., 2008; Naoumkina *et al*., 2007). Metabolite identification using liquid chromatography coupled with tandem mass spectrometry (LC‐MS/MS) and ultra‐performance liquid chromatography coupled to electrospray ionization quadrupole time‐of‐flight MS (UPLC‐ESI‐q‐Tof‐MS), we showed that the major isoflavonoids accumulated in wild‐type *M. truncatula* hairy roots were formononetin 7‐*O*‐glucoside malonate, formononetin 7‐*O*‐glucoside, glycitein, 4′,6‐dihydroxy aurone, genistein and biochanin A, as well as other isoflavonoid glycosides and malonates (Figure 3). These hairy roots also accumulated flavonols and flavanones including kaempferol 3‐*O*‐rutinoside, quercetin, 6, 7, 4‐trihydroxyflavone, 7, 3, 4, 5‐tetrahydroxyflavone, naringenin *4*′‐*O*‐glucoside, dihydroxyquercetin and luteolin *5*′, *7*‐*O*‐glucoside. The comparison of flavonoid profiles in *M. truncatula* hairy roots over‐expressing *MtPAR* with respect to GUS control lines showed that many substantial changes in major (iso)flavonoid metabolites (Figure 3). Regarding increase of flavonoid content, *MtPAR* over‐expression resulted in more than 10‐fold increase in contents of epicatechin 3′‐*O*‐glucoside and more than fivefold increase in epicatechin aglycone with respect to GUS control lines. The contents of two flavonols, kaempferol 3‐*O*‐rutinoside and quercetin were increased by more than twofold and 100‐fold, respectively (Figure 3c). The other flavonoids such as 6, 7, 4‐trihydroxyflavone, 7, 3, 4, 5‐tetrahydroxyflavone, luteolin‐5′, 7‐*O*‐diglucoside and naringenin 4′‐*O*‐glucoside were also increased by fivefold, fourfold, sevenfold and twofold, respectively, in *MtPAR* hairy roots compared to GUS controls (Figure 3c). On the other hand, we observed significant reductions of the major isoflavonoids: the content of formononetin 7‐*O*‐glucoside malonate was reduced in *MtPAR* hairy roots by more than 10‐fold compared with GUS control, formononetin 7‐*O*‐glucoside reduced by ~1.7‐fold, glycitein reduced by ~1.7‐fold and 4′,6‐dihydroxy aurone (more than 10‐fold), genistein (~1.7‐fold), genistin (more than twofold) and biochanin A (more than sixfold) (Figure 3d).

These metabolite profiling data are consistent with microarray and qRT–PCR data on hairy roots over‐expressing *MtPAR* with respect to control lines confirmed dramatic down‐regulation in several isoflavonoid biosynthetic genes, such as *isoflavone O‐methyltransferases*,* isoflavone glycosyltransferases* and isoflavonoid specific *chalcone synthase* (*CHS*) (Table S2). Further qRT–PCR experiments confirmed that *MtPAR* over‐expression resulted in up‐regulation of PA‐specific genes, while isoflavonoid specific genes, such as *CHI1, CHI3*,* IFS* and *IFR,* were down‐regulated (Figure 3d,e).

### Ectopic expression of MtPAR repressed isoflavone accumulation in soybean hairy roots

**Figure 4:**
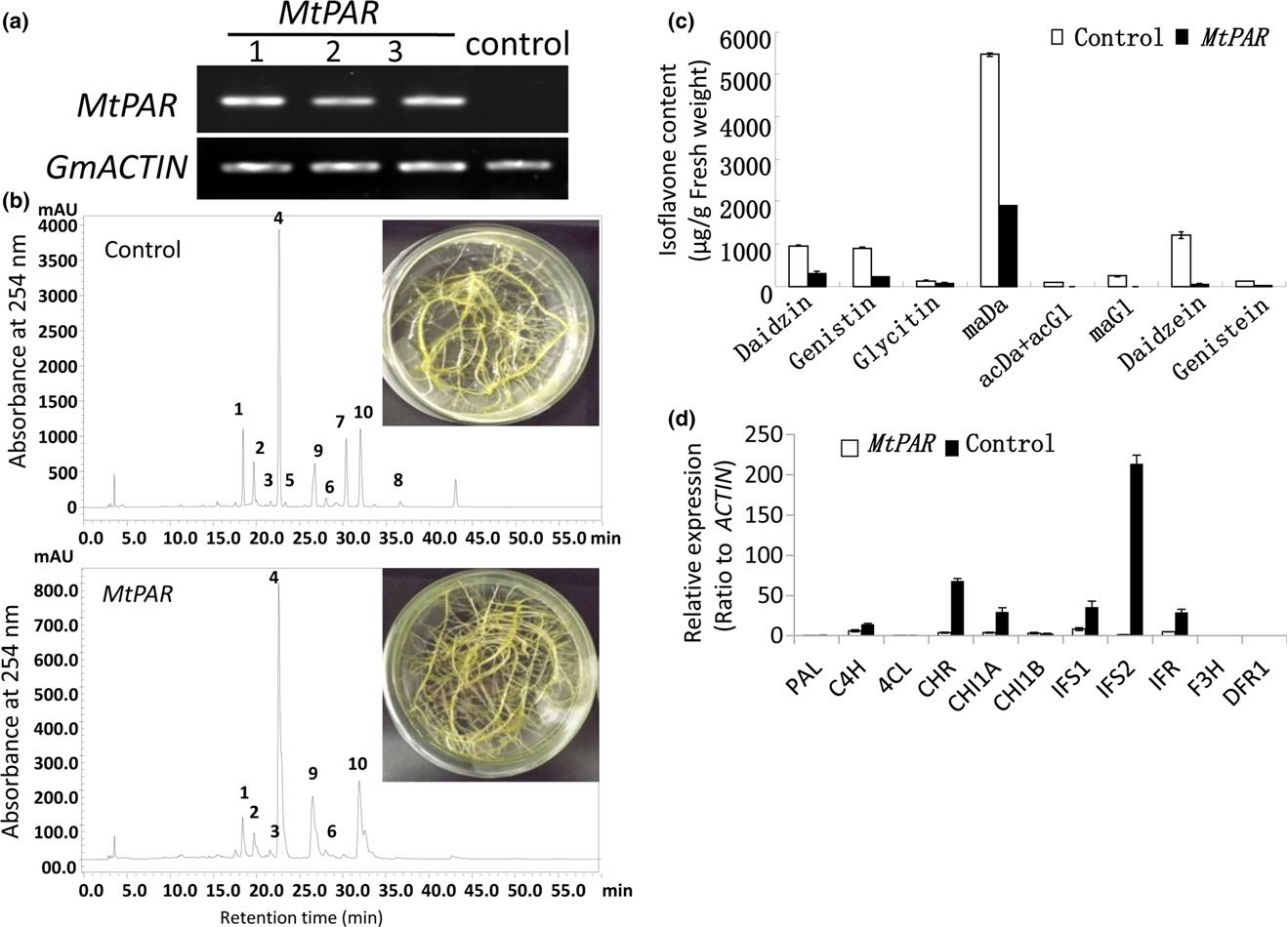
*MtPAR* represses isoflavonoid production in soybean (*Glycine max*) hairy roots. (a) Semiquantitative RT‐PCR showing *MtPAR* overexpression in soybean hairy roots. (b) HPLC chromatographs of major isoflavonoids in soybean hairy roots expressing *MtPAR* and *GUS* control. Peak numbers correspond to 1‐daidzin, 2‐glycitin, 3‐genistin, 4‐malonyl daidzin, 5‐acetyl daidzin and acetyl glycitin, 6‐malonyl genistin, 7‐daidzein, 8‐genistein, 9/10‐flavonols. (c) Accumulation of major isoflavonoids in transgenic hairy roots. (d) Expression profiles of isoflavonoid biosynthetic genes in soybean transgenic hairy roots. Relative expression was calculated from qRT‐PCR data with respect to *ACTIN*. Data are expressed as mean ± SD from at least three independent experiments.

**Figure 5:**
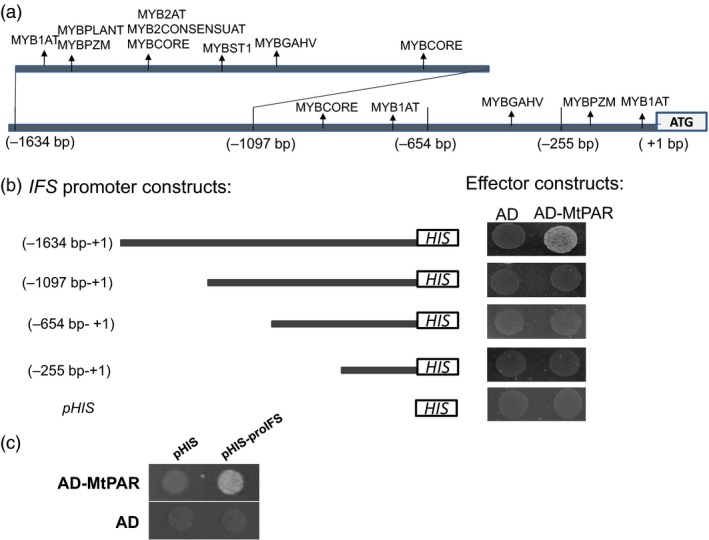
*MtPAR* binds to *IFS* promoters to represses isoflavonoid production in legume (a) *cis*‐elements in soybean *IFS* promoter that are targeted by MYB transcription factor in prediction (b) Analysis of *GmIFS2* promoter for MtPAR binding activity. Various *GmIFS2* promoter deletion constructs were made to test MtPAR binding regions in a yeast one‐hybrid assay. *The cis*‐elements that may be bound by MYB transcription factors in *GmIFS2* promoter and the upstream sites for construction of various deletion mutants are listed. (c) Yeast one‐hybrid assay on MtPAR binding to *MtIFS1* promoter. The promoters of *MtIFS1 or GmIFS2* were cloned in front of the HIS3 gene and integrated into Y1H yeast strains to generate reporter strains. The reporter strains were transformed with the effector *MtPAR* construct, and growth was recorded in the presence or absence of His as well as 3‐AT at 30 mm supplemented to SC medium. Photographs are representatives from three biological replicates.

To explore that how MtPAR repressed isoflavone biosynthesis, we examined the impact of *MtPAR* over‐expression on isoflavones in soybean hairy roots. Soybean hairy roots primarily synthesize high levels of isoflavonoids and undetectable levels of PAs and anthocyanins (Gutierrez‐Gonzalez *et al*., 2010). Over‐expression of *MtPAR* in soybean hairy roots resulted in severe suppression of isoflavonoid production, although visible accumulation of anthocyanins and PAs could hardly be seen (Figure 4a–c). All major isoflavonoids such as malonyl daidzin (maDa), daidzin, glycitin, genistin and daidzein were reduced by 2.5‐fold, threefold, twofold, fourfold and eightfold, respectively (Figure 4c). Consistently, the expression of flavonoid genes was down‐regulated in the *MtPAR* transgenic hairy roots compared with GUS control (Figure 4d). As *MtPAR* over‐expression down‐regulated *IFS* expression in both *M. truncatula* and soybean hairy roots, we analysed promoter sequences of different *IFS* genes from several legumes and identified several *cis*‐regulatory elements that have been identified as MYB transcription factor binding sites (Table S3, Figure 5a).

To test whether *MtPAR* can directly bind *GmIFS* promoter region, we performed a yeast one‐hybrid assay to test interaction between MtPAR protein and *GmIFS2* promoter sequence (~1.6 kb). MtPAR did bind to *GmIFS2* promoter and may thus repress isoflavone accumulation in soybean hairy roots (Figure 5b). To further validate the interaction and determine the *cis*‐elements involved in the MtPAR‐DNA interaction, this ~1.6 kb fragment upstream to *GmIFS2* gene was constructed into various deletion fragments into pHIS2 vector and then transformed into Y187 strains co‐expressing *MtPAR* in reading frame with GAL4 activation domain. Only full‐length fragment of ~1.6 kb showed binding activity, other fragments from −1097 bp to start codon were not involved in the MtPAR binding (Figure 5b). The more promoter region from −1097 to −1634 bp contains MYB binding *cis*‐elements such as MYBCORE and MYB2AT, which are likely sites for MtPAR interaction. As expected, MtPAR also bound to *MtIFS1* promoter of ~1.5 kb, as the promoter regions of *Medicago truncatula MtIFS1* are similar to *GmIFS2* (Figure 5c).

### MtPAR interacts with MtTT8 and MtWD40‐1 to form a ternary complex

**Figure 6:**
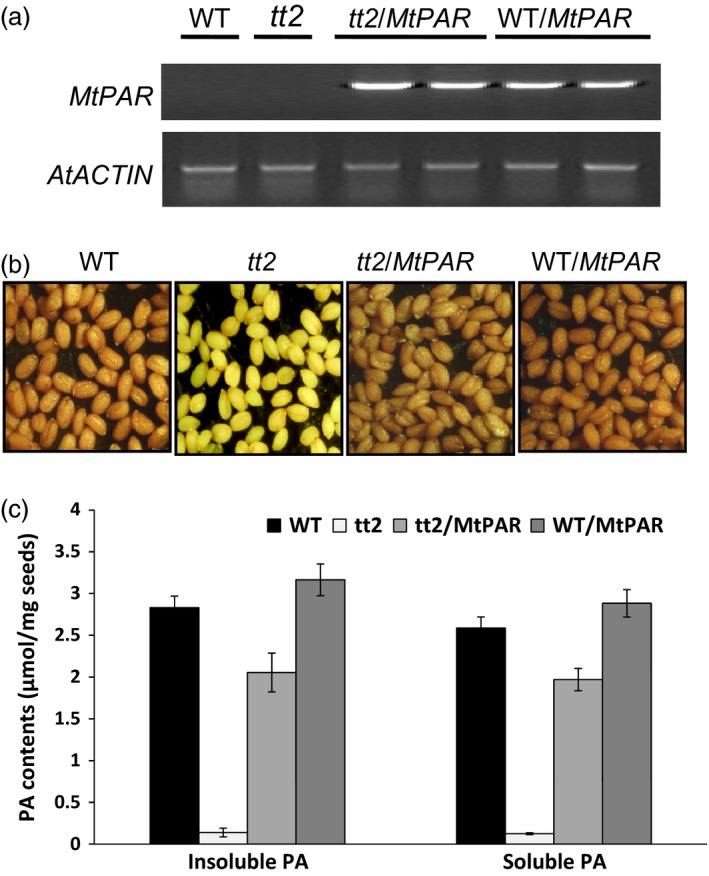
Complementation of the PA deficiency phenotype in *tt2* mutant seeds by *MtPAR*. (a) Semiquantitative RT‐PCR showing *MtPAR* overexpression in *tt2* mutant and wild‐type Arabidopsis (Col‐0) seeds. (b) Seed coat phenotypes of wild‐type (WT), *tt2*,* MtPAR*/*tt2*,* MtPAR*/WT seeds. (c) Quantification of soluble and insoluble PAs in seeds of Arabidopsis transgenic lines. Data represent mean from the three biological replicates with their respective SD.

We then conducted genetic complementation of Arabidopsis *tt2* mutant using a *35S::MtPAR*. The expression of *MtPAR* in *tt2* mutant seeds was confirmed by RT–PCR (Figure 6a). The *tt2* mutant seeds displayed a pale yellow colour compared to the brown colour of wild‐type seeds (Figure 6b). Overexpression of *MtPAR* gene in *tt2* plants restored the seed colour phenotype, although the complemented seeds were not as brown as the wild‐type seeds (Figure 6b). Analysis of soluble and insoluble PA contents in seeds confirmed that *MtPAR* overexpression partially restored PA production in *tt2* mutant seeds to wild‐type levels (Figure 6c). Moreover, we observed that the overexpression of *MtPAR* in wild‐type plants resulted in an increase of total PA content (~22%) in transgenic seeds, suggesting that MtPAR shares similar functions as AtTT2 (Figure 6c).

**Figure 7:**
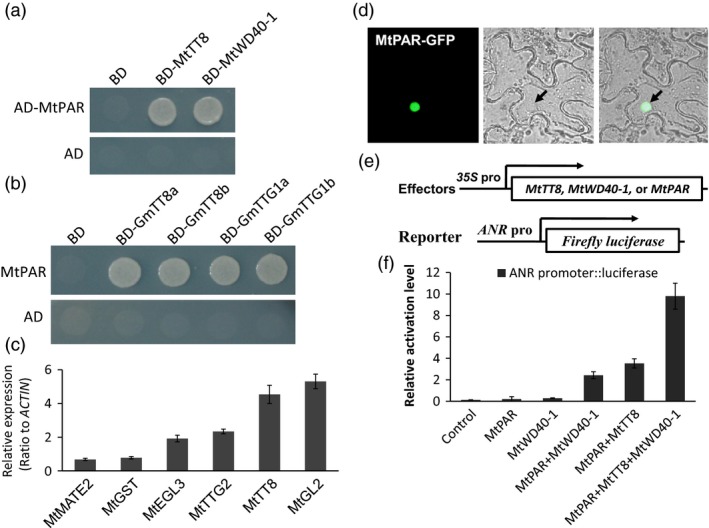
MtPAR interaction with bHLH and WD40 transcription factors for activation of *ANR* promoter. (a) Y2H assay between MtPAR with *Medicago truncatula* MtTT8 and MtWD40 proteins. (b) Y2H assay between MtPAR with *Glycine max* TT8 and WD40 proteins. (c) Induction of downstream genes of bHLH‐MYB‐WD40 (MBW) complex in *M. truncatula* hairy roots over‐expressing *MtPAR* by q‐RT‐PCR. (d) Subcellular localization of MtPAR‐GFP in tobacco epidermal cells. (e) Effector constructs and ANR promoter driven reporter gene construct for transient expression assays. (f) *Trans*‐activation assay of *ANR* promoter activity in Arabidopsis leaves by transient expression of *MtPAR* together with other effector genes. Luciferase activities are expressed as relative activity compared to the value obtained with the control, All luciferase activity data were normalized with Renilla LUC reporter construct alone. Data are expressed as mean ± SD from at least three biological replicates.

We further explored the mechanism by which *MtPAR* promotes PA biosynthesis, by examining if MtPAR could interact with MtTT8 and MtWD40‐1 to form a regulatory complex (Albert *et al*., 2014). Using yeast two‐hybrid (Y2H) system, in which the GAL4‐binding domain (BD) fused with coding sequences of MtTT8 and MtWD40‐1 and activation domain (AD) fused with MtPAR coding sequence, we tested protein–protein interactions in yeast. We observed interactions between MtPAR with both MtTT8 and MtWD40‐1 (Figure 7a). As MtPAR possesses a conserved function in other legumes, we tested MtPAR interaction with soybean orthologs of MtTT8 and MtWD40‐1. We identified two orthologs of MtTT8 in soybean, GmTT8a (Glyma10G03140.2) and GmTT8b (Glyma02G16670.2), and two orthologs of MtWD40‐1, GmWD40a (Glyma16G04930), GmWD40b (Glyma06G14180). We also observed clear interactions between MtPAR and GmTT8s and GmWD40s (Figure 7b). In Arabidopsis and Medicago, the MBW ternary complexes have been showed to activate expression of downstream transcription factors such as *AtEGL3*,* AtGL2*,* AtTT8* and *AtTTG2* (Payne *et al*., 2000; Liu *et al*., 2014; Xu *et al*., 2013, 2014). Transcriptomic data showed up‐regulation of corresponding genes *MtEGL3* (Medtr8g098275), *MtGL2* (Medtr2g101720), *MtTT8* (Medtr1g072320) and *MtTTG2* (Medtr7g100510) in Medicago hairy root lines over‐expressing *MtPAR* (Figure 7c and Table S1).

### MtPAR activate *ANR* promoter in the presence of MtTT8 and MtWD40‐1

In a subcellular localization assay, MtPAR‐GFP signal was clearly observed in the nucleus of the epidermal cells in tobacco leaves (Figure 7d), indicating its possible function as a MYB transcription factor. To examine MrPAR‐activated PA biosynthetic genes through the MBW complex, we conducted promoter *trans*‐activation assays. When *ANRpro::luciferase* reporter construct was co‐bombarded with *35S‐*driven *MtPAR* with *MtTT8* and *MtWD40‐1* effector constructs into Arabidopsis leaves (Figure 7e), *ANR* promoter‐*luciferase* reporter by around nine‐folds compared with empty vector controls (Figure 7f), while combination of *MtPAR, MtWD40‐1*, or *MtPAR* showed no such dramatic activation. Therefore, MtPAR forms a ternary complex with MtWD40‐1 and MtTT8 to activate PA biosynthesis.

### MtPAR and MtLAP1 additively promote PAs in alfalfa hairy roots

**Figure 8:**
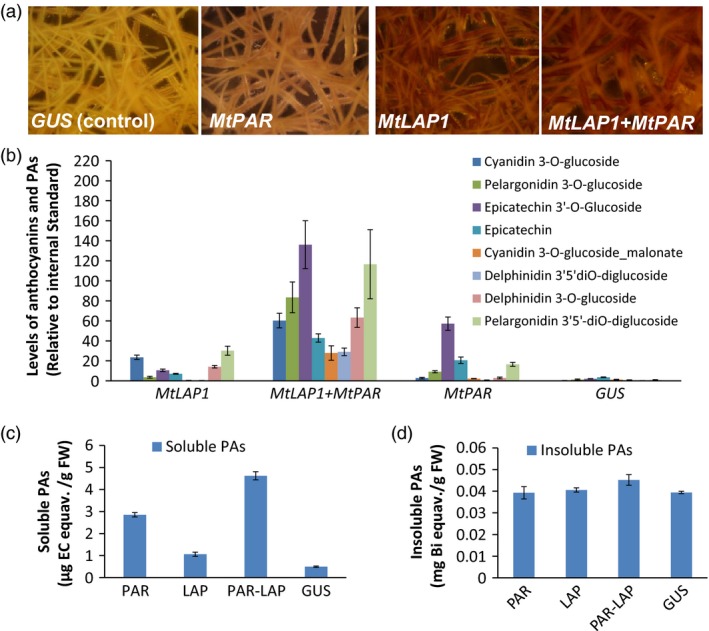
Co‐expression of *MtPAR* and *MtLAP1* in alfalfa (*Medicago sativa*) hairy roots. (a) Pictures of alfalfa hairy roots lines expressing *MtPAR*,* MtLAP1*,* MtLAP1*‐*MtPAR* and *GUS* (control). (b) Profiling of major metabolites in alfalfa hairy roots over‐expressing *MtPAR*,* MtLAP1*,* MtPAR*‐*MtLAP1* and *GUS*. (c, d) Accumulation of soluble (c) and insoluble (d) PAs in different transgenic hairy roots. Data represent mean of at least three biological replicates ± SD. Photographs are representatives of at least five lines. Data are expressed as mean ± SD from at least three biological replicates.

As *MtPAR* promoted PA biosynthesis at the expense of decreases in isoflavonoid and anthocyanins production (Figure 1), the limited PA production in *MtPAR* transgenic alfalfa shoots might be due to the limitation in precursor anthocyanidins as usually alfalfa does not synthesize as much anthocyanins as hairy roots of *M. truncatula*. We therefore validate if the anthocyanidin precursors are the limiting factor for MtPAR‐promoted PA production. When both *MtPAR* and *MtLAP1* were over‐expressed in alfalfa hairy roots, individually and combined, alfalfa hairy roots expressing *GUS* (control lines) did not synthesize visible anthocyanin pigments (Figures 8a and S3). However, ectopic expression of *MtLAP1* resulted in a massive accumulation of anthocyanins (i.e. cyanidin 3‐*O*‐glucoside, delphinidin 3, 5‐*O*‐diglucoside and pelargonidin 3, 5‐*O*‐diglucoside) and a slight increase of insoluble PA‐like substances in hairy roots, as observed by Peel *et al*. (2009)(Figures 8b–d and S3, S4). Ectopic expression of *MtPAR* in alfalfa hairy roots stimulated the accumulation of PAs, including epicatechin 3′‐*O*‐glucoside and insoluble PAs, and slightly increased anthocyanins, mainly pelargonidin 3, 5‐*O*‐diglucosides (Figures 8b–d and S3, S4). However, co‐expression of both *MtPAR* and *MtLAP1* significantly enhanced the accumulation of PAs and anthocyanins (Figures 8b and S3, S4), demonstrating an additive activation effect of two genes on PA and anthocyanin biosynthesis. We observed a stronger 4‐dimethylaminocinnamaldehyde (DMACA) staining, which specifically stain PA monomer and polymers, than GUS control lines when *MtPAR* was expressed, staining became even much stronger when both *MtPAR* and *MtLAP1* were expressed (Figure S5).

Metabolite profiling using UPLC‐ESI‐q‐TOF‐MS confirmed that *MtLAP1* hairy roots showed higher production of several anthocyanins, such as cyanidin 3‐*O*‐glucoside, pelargonidin 3‐*O*‐glucoside and their malonates, as well as flavonoids, such as genistein, apigenin, naringenin, kaempferol 3‐*O*‐rutinoside, luteolin 4′‐*O*‐glucoside, and tri‐, tetra‐pentahydroxyl flavone, such as 6, 7, 4′‐trihydroxyflavone, 3, 6, 2′, 3′‐tetrahydroxyflavone and 3, 7, 3′, 4′, 5′‐pentahydroxyflavone. *MtPAR* hairy roots produced more PAs and slightly higher amount of anthocyanins and flavonols. Co‐expression of *MtPAR* and *MtLAP1* significantly increased levels of all these anthocyanins, PAs, flavanones and flavonols, as well as some saponins (Figure S7). However, the levels of epicatechin and epicatechin 3′‐*O*‐glucoside and PAs, as well as anthocyanins, were among the most drastically enhanced by *MtPAR* and *MtLAP1* co‐expression, suggesting an additive effect of MtPAR and MtLAP1 (Figures 8b and S7).

Transcriptomic analysis using qRT–PCR confirmed the additive effect of *MtPAR* and *MtLAP1* on up‐regulation of genes involved in both PA and anthocyanin biosynthesis. *PAL*,* C4H*,* 4CL, CHS, F3H*,* ANS* and *ANR* were up‐regulated to different degrees in *MtPAR*,* MtLAP1* and *MtLAP1‐MtPAR* hairy roots with much higher expression in *MtLAP1‐MtPAR*‐co‐expression lines (Figure S7a).

### Alfalfa plants co‐expressing *MtPAR* and *MtLAP1* produced more PAs

**Figure 9:**
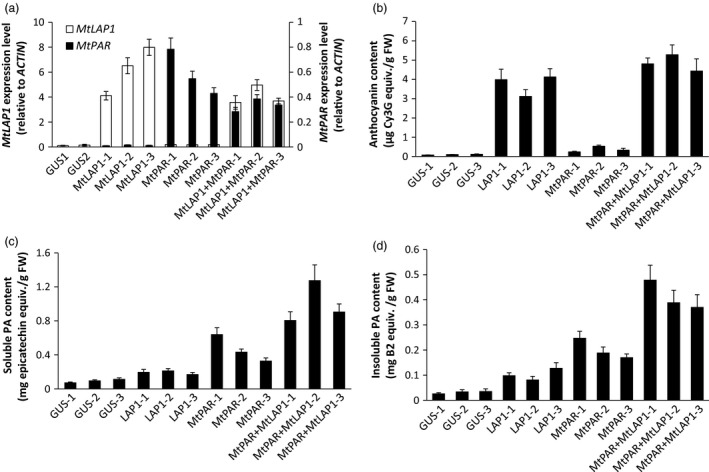
Enhanced production of PAs in alfalfa plants co‐expression of *MtPAR* and *MtLAP1*. Values represent averages ± SD from at least three biological replicates. (a) *MtPAR* and *MtLAP1* transcript levels in aerial part (stems + leaves) of transgenic alfalfa lines. (b) Anthocyanin contents in *MtPAR*,* MtLAP1*,* MtPAR‐MtLAP1* and *GUS* transgenic alfalfa lines. (c) Soluble PA contents in different transgenic alfalfa lines. (d) Insoluble PA contents in different transgenic alfalfa lines. Data are expressed as mean ± SD from at least three biological replicates.

As *MtLAP1* and *MtPAR* showed a synergic effect on PA production in alfalfa hairy roots, we generated stable transgenic alfalfa plants co‐expressing *MtLAP1* and *MtPAR*. We crossed the 35S::*MtLAP1* and 35S::*MtPAR* transgenic alfalfa lines previously generated in Peel *et al*. (2009) and Verdier *et al*. (2012). Of more than 20 alfalfa transgenic lines co‐expressing *MtLAP1* and *MtPAR*, we selected transgenic lines displaying the highest expression of both transgenes (Figure 9a). Analysis of anthocyanins and total PAs in shoots revealed that ectopic expression of both genes slightly increased the anthocyanin content (Figure 9b) but significantly increased the total PA production compared to transgenic lines over‐expressing *MtPAR* alone (Figure 9c,d). qRT–PCR analysis suggested that the increase of PA production in these transgenic lines was consistent with the increase of expression of several enzymes of the flavonoid pathway, which were highly up‐regulated in these lines, including some PA‐specific enzymes such as *ANR* (Figure S7b). The enhanced production of PAs in leaves and stems was further confirmed by tandem mass spectrometry, which detected significant amounts of epicatechin monomers, such as epicatechin and epicatechin 3′‐*O*‐glucoside, as well as B1 dimmers and larger oligomers (Figure S8).

## Discussion

To enhance a moderate PA production in legume forage alfalfa, we had tried to understand the transcriptional regulation of PA biosynthesis in model legume Medicago (Dixon *et al*., 2005; Verdier *et al*., 2012). We had further explored the mechanism by which MtPAR promotes PA production, and furthermore, we optimized metabolic engineering of PA in alfalfa with MrPAR and MtLAP1. We showed that at least two distinct but connecting mechanisms exist for MtPAR to promote PA production in legume: first, repressing isoflavone pathway to direct chalcone/naringenin precursors flux towards flavonoid biosynthesis, and secondly, redirecting immediate precursors, anthocyanidins, from anthocyanin pathway to PA biosynthesis. Based on these results, we proposed that MtPAR specifically regulates PA productions, and it may achieve bigger promotion on PA production if more anthocyanidin precursors were accumulated in alfalfa. In a proof‐of‐concept trial, we designed the co‐transformation of two genes, *MtPAR* and *MtLAP1*, into transgenic alfalfa. Indeed, an additive effect by two genes in promoting PA production in alfalfa shoots was observed, and a substantial PA production was produced in transgenic alfalfa.

### Ectopic expression of MtPAR directly represses isoflavone biosynthesis

Little is known about specific transcription factors activating or repressing isoflavone synthesis, and about regulatory mechanisms for isoflavone biosynthesis (Shelton *et al*., 2012; Li and Dhaubhadel, 2012). We consistently observed a reduction in isoflavone content in *M. truncatula*, alfalfa and soybean hairy roots over‐expressing *MtPAR* (Figures 2a, 3d and 4c). Similarly in these transgenic lines, isoflavone biosynthetic genes, such as *IFS or IFR*, were down‐regulated, whereas PA biosynthetic genes were up‐regulated (Figures 2d, 2e and 4d). These results suggest that *MtPAR* could directly or indirectly repress isoflavone biosynthesis, independent of anthocyanidin precursors or anthocyanins. *IFS* is one of the key genes in the branching point for isoflavone, flavanone and dihydroflavonol biosynthesis pathways (Figure 1). An *in silico* analysis of promoters of different *IFS* genes from various legumes species (i.e. *M. truncatula*, soybean, common bean and alfalfa) revealed presence of many *cis*‐elements known to be targeted by MYB transcription factors (Table S3). We revealed MtPAR protein binding to *GmIFS2* and *MtIFS1* promoter sequence using yeast one‐hybrid assay. We further determined the binding site of MtPAR to the ~600 pb region of *GmIFS2* promoter where many MYB factor binding *cis*‐elements presents in high density (Figure 5a–f). As MtPAR does not to have a predicted repressor domain in C‐terminus, we postulated that MtPAR repressed isoflavone biosynthesis, probably through binding to *IFS* promoter, by either competing with isoflavone MYB activator or other mechanisms, which are worth further investigation. In fact MtPAR not only repressed *IFS*, but also repressed other isoflavone biosynthetic genes. *IFS* and other isoflavone genes in Medicago can be regulated by unknown MYB activators, more likely in a complex similar to MBW that regulates PA biosynthesis (Shelton *et al*., 2012). MYB activator of isoflavone biosynthesis may interact with other components to form an active complex for activation of isoflavone biosynthesis by binding to *cis*‐elements in the promoters of *IFS* or isoflavone genes (Li and Dhaubhadel, 2012). Additionally, MtPAR also likely impacted the negative effect on isoflavone production indirectly by transrepression (Ohta *et al*., 2001). MtPAR may repress isoflavonoids indirectly by inducing the expression of an isoflavone repressor, just like MBW complex activate MtMYB2, a repressor of PA and anthocyanin biosynthesis (Jun *et al*., 2015). Nevertheless, our results suggest that MtPAR could re‐orientate the chalcone or naringenin–chalcone precursor flux into anthocyanin and flavonol pathways by repressing isoflavone pathway genes. This can significantly promote flavonoid biosynthesis as isoflavones are prevalent products in legume plants.

### Ectopic expression of *MtPAR* does not dramatically affect anthocyanin genes

The biosynthetic pathways leading to accumulation of anthocyanins and PAs share common anthocyanidin precursors (i.e. leucoanthocyanidins and anthocyanidins). ANR and UGTs are regarded as the key enzymes competing for anthocyanidin precursors for production of PAs and anthocyanins, respectively, and UGTs may have higher catalysing efficiency than ANR, and the expression levels of *UGT* are higher than that of *ANR* (Lee *et al*., 2005; Zhao *et al*., 2011). In our study, we observed a negative correlation between PA and anthocyanin accumulation in hairy roots, supporting a competition for anthocyanidin precursors between these two pathways. Ectopic expression of *MtPAR* did not substantially alter the expression of anthocyanin biosynthesis‐related *UGT*s or anthocyanin transporter gene *MtMATE2*, but promoted the expression levels of flavonol pathway genes, such as *flavonols synthase* (*FLS)*, and PA‐specific biosynthesis genes, such as *ANR, UGT72L1,* and the epicatechin transporter *MtMATE1* (Table S2). We concluded that *MtPAR* more specifically activated PA biosynthesis, not only by activation of whole anthocyanidin biosynthetic genes, but also redirect the anthocyanidin precursors into PA biosynthesis in transgenic alfalfa hairy roots and plants (Figures 8b and 9b). Thus, MtPAR negatively affected anthocyanin accumulation, not by affecting their synthetic genes, but by redirecting anthocyanidin precursors into PA pathway. This result suggests that the anthocyanin biosynthesis is predominant over PA biosynthesis in our models, and shifting the anthocyanidin precursors from anthocyanin to PA pathway is essential for PA production.

### MtPAR directly interacts with bHLH and WD40 to regulate PA biosynthesis

The previous study has shown that MtPAR alone could not bind to the promoter of *ANR* gene; but it directly binds to *MtWD40‐1* promoter and regulates a subset of PA biosynthetic genes (Verdier *et al*., 2012). However, overexpression of *MtWD40‐1* alone failed to increase PA production, although MBW transcription factors are indispensible in regulating PA biosynthesis (Lepiniec *et al*., 2006; Pang *et al*., 2009; Xu *et al*., 2013, 2014; Albert *et al*., 2015). Here, we proved that MtPAR interacts with MtTT8, MtWD40‐1 to form a MtPAR‐MtWD40‐1‐MtTT8 MBW complex, which can *trans*‐activate the promoter of *ANR* and activating PA biosynthesis (Figure 7a–f). MtPAR interaction with MtWD40‐1 and MtTT8 should also activate flavonol and PA biosynthetic genes, such as *FLS*,* DFR*,* ANS*,* UGT72L1* and *MtMATE1*.

Ternary MBW complexes are involved in different processes such as biosynthesis of seed PAs and anthocyanins, leaf trichome formation and root hair patterning (Albert *et al*., 2014, 2015; Debeaujon *et al*., 2003; Johnson *et al*., 2002; Lepiniec *et al*., 2006; Nesi *et al*., 2000, 2001). It has been demonstrated in Arabidopsis that PAP1‐TT8 (GL3)‐TTG1 complex controls anthocyanin production, and TT2‐TT8‐TTG1 complex controls PA production (Baudry *et al*., 2004; Lepiniec *et al*., 2006; Xu *et al*., 2013, 2014), Medicago MtLAP1 can also form a complex with MtTT8 and MtWD40‐1 to activate anthocyanin biosynthesis (Peel *et al*., 2009; Li *et al*., 2015). MtMYB5 and MtMYB14 interact in the presence of MtTT8 and MtWD40‐1 to form the MBW regulatory complex in regulating PA biosynthesis (Liu *et al*., 2014). MtPAR and MtLAP1 may also form complexes with MtTT8 and MtWD40‐1 to activate PA production or isoflavone repressors. Recent study indicated that a R2R3 MYB transcription factor MtMYB2, which is activated by both MtPAR, MtMYB5 and MtMYB14, interact to form a complex to compete with MtMYB5 and MtMYB14 for interaction with MtTT8, and thereby represses PA biosynthesis in a temporal–spatial manner (Jun *et al*., 2015). Therefore, it is likely that overexpression of MtPAR and MtLAP1 may activate isoflavone repressors and thus cause a repression indirectly (Kang *et al*., 2009). Like MtMYB5 and MtMYB14 in MBW complex (Liu *et al*., 2014), it also likely that MtLAP1 and MtPAR interact in the presence of MtTT8, which can dimerize to bridge the interaction between MtLAP1 and MtPAR. Thus, MtLAP1 co‐expression with MtPAR releases the repression of MtPAR from repressing isoflavone biosynthesis.

### Combination of MtPAR and MtLAP1 as a tool for engineering PA in legumes

The biosynthesis of both PAs and anthocyanins require anthocyanidins as precursors; it has been proposed that competition for anthocyanidin precursors may be one of the major obstacles in metabolic engineering of PAs (Lepiniec *et al*., 2006; Li et al., 2015). In several studies, preferential production of anthocyanins rather than PAs suggests that the two enzymes, UGTs and ANRs, are not equally competitive regarding precursor availability (Lee *et al*., 2005; Marinova *et al*., 2007; Shelton *et al*., 2012; this study). Overexpression of *MtPAR* can clearly enhance the ANR expression and competition for anthocyanidin precursors, and thus directed more anthocyanidins for PA production.

Besides this, MtPAR also surprisingly and significantly repressed isoflavone biosynthesis; the repression directs even more chalcone and flavonone precursors flux into PA and flavonol biosynthesis. Because isoflavones are the major flavonoids, the isoflavone repression by MtPAR may make much bigger contributions to PA production than redirecting anthocyanidin precursors into PAs, as supported by both transcriptomic and metabolomic analysis. In supporting this metabolic flux shift, MtPAR differentially regulates two MATE transporters, *MtMATE1* and *MtMATE2*. It up‐regulated the epicatechin 3′‐*O*‐glucoside transporter gene *MtMATE1* without affecting the expression of anthocyanin transporter *MtMATE2* in hairy roots (Zhao and Dixon, 2009; Zhao *et al*., 2011; Zhao, 2015). Such up‐regulation of whole metabolic pathway, modification and transport processes strongly supports the note that MtPAR recruited all metabolic flux mechanisms to promote PA production.

An effective bloat protection PA level is about 20 mg/g dry weight (Verdier *et al*., 2012; Hancock *et al*., 2012). The insufficient PA production in shoots of *MtPAR* transgenic alfalfa may have many reasons, such as low expression of *MtPAR* and limitation in anthocyanidins precursors. *MtLAP1* over‐expression induced a massive production of anthocyanins, but not PA accumulation in alfalfa (Peel *et al*., 2009; Figure S3). By over‐expressing *MtLAP1* in combination with *MtPAR*, we observed an additively promoted PA production (Figure 9c,d). In alfalfa hairy roots co‐expressing *MtLAP1* and *MtPAR*, overall isoflavones decreased while anthocyanins and PA production dramatically increased. As a proof of concept, transgenic alfalfa plants coexpressing *MtLAP1* and *MtPAR* produced a substantially improved PAs. The additive effect likely resulted from the combined effects, including the increased anthocyanidin precursor poor by *MtLAP1* overexpression, the repression of isoflavone pathway and the activation of PA biosynthesis by *MtPAR* overexpression. These effects together force metabolic flux flowing into PA biosynthesis in *MtLAP1* and *MtPAR* co‐transformed alfalfa.

In conclusion, we provided that MtPAR reduced flavonoid precursor flux into isoflavone pathway but direct these precursors flowing into anthocyanins and flavonols and then redirects immediate precursor anthocyanidins from the parallel anthocyanin pathway to PA biosynthesis. We further successfully improved the performance of metabolic engineering of PAs in alfalfa with MtPAR with MtLAP1. The study provides new insight into regulatory mechanisms of PAs in complex metabolic network through regulatory protein complexes and *trans*‐activation of key structural genes. This result also provides an efficient strategy for metabolic engineering of PA in legume forage, which might be able to make a breakthrough leading to a successful breeding of bloat‐safe alfalfa varieties for forage production and safe grazing.

## Experimental procedures

### Ectopic expression of *MtPAR* in Medicago and soybean hairy roots

The open reading frame (ORF) of *MtPAR* was cloned into the entry vector pENTR/D/TOPO or pDONOR221 (Invitrogen, Rockville, MD, USA). After sequencing confirmation, the *MtPAR* ORF was recombined into a destination vector, pB7WG2D and pB2GW7 using the LR clonase reaction (Invitrogen). pB7WG2D‐MtPAR (for hairy roots) and pB2GW7‐MtPAR (for plants) were used for *M. truncatula*,* M. sativa* (alfalfa) and *Glycine max* (soybean) hairy roots or plant transformation, as well as Arabidopsis transformation. The GUS gene was also recombined into the entry/destination vectors to be used as control.

To generate *M. truncatula* and soybean hairy roots, pB7WG2D vectors harbouring *MtPAR* or *GUS* sequences were transformed into *Agrobacterium rhizogenes* strains ARqua1 and K599 by electroporation for *M. truncatula* and soybean transformation, respectively (Quandt *et al*., 1993). Transformed colonies were grown on LB‐agar medium at 28 °C, with spectinomycin and streptomycin for selection. After PCR validation, Agrobacteria containing the constructs were used to transform *M. truncatula* (cv. Jemalong A17) or soybean (c.v. Tianlong No.1) according to Boisson‐Dernier *et al*. (2001). The resulting hairy roots were maintained on B5 agar media in Petri dishes supplied with 7.5 mg/L phosphinothricin (ppt) under fluorescent light (140 μE/m^2^ s^1^) with a 16‐h photoperiod and were subcultured every 20 days onto fresh media. Screening of transformed hairy root was performed by observation of GFP signal, which was used as transformation marker, by staining with DMACA reagent for presence of PAs and by qRT–PCR analysis to detect and quantify the *MtPAR* transcript level.

For alfalfa hairy root transformation, ecotype RGSD4 was used for transformation with *A. rhizogenes* strain ARqua1 harbouring the different constructs pB7WG2D‐MtPAR or pB7WG2D‐MtLAP1 (Peel *et al*., 2009). The transformation protocol was described previously (Verdier *et al*., 2012). Arabidopsis stable transformation was conducted using floral dipping method described previously (Clough and Bent, 1998). *MtPAR‐pB2GW7* was transformed into *A. tumefaciens* strain LBA4404, which was used for transformation of Arabidopsis *tt2* mutant line (SALK line CS83).

### Establishment of Alfalfa transgenic plants co‐expressing *MtPAR* and *MtLAP1*

Alfalfa transgenic lines over‐expressing *MtPAR* and *MtLAP1* were generated by Peel *et al*. (2009) and Verdier *et al*. (2012). The two transgenic lines were crossed together to obtain Alfalfa plants over‐expressing both *MtPAR* and *MtLAP1*. Over‐expression of both genes was confirmed by qRT–PCR (Figure 8a), plants with the highest expression for both genes were selected for further studies.

### RNA extraction, microarray and qRT–PCR analysis

Total RNA from Alfalfa and soybean was isolated using a modified CTAB method (Verdier *et al*., 2012) from hairy roots and using Trizol reagent from alfalfa shoots according to the manufacturer's instructions (Invitrogen). Ten μg of total RNA from each sample were DNAse treated (Turbo DNase; ThermoFisher Scientific,Waltham, MA, USA) and purified (RNA easy Min Elute Cleanup kit; QIAgen, Valencia, CA, USA), according to manufacturer's instructions. Five hundred nanograms of purified RNA for each of the three biological replicates was used for probe synthesis using a Gene Chip 3′ IVT express kit, according to manufacturer's instructions (Affymetrix, Sacramento, CA, USA). Hybridization of probes to Affymetrix Gene Chip Medicago genome arrays, normalization and differentially expressed gene analysis were carried out as described previously (Irizarry *et al*., 2003; Verdier *et al*., 2012). Data are publicly available in Array Express database (E‐MEXP‐3485).

Quantitative reverse transcription–polymerase chain reaction analysis was performed using cDNA synthesized by Super Script III from 2 μg of DNAse‐treated RNA, according to manufacturer's instructions (Invitrogen). Amplification reactions were performed in 5 μL final volume containing 2.5 μL of Power SYBR Master mix (Applied Biosystems), 1 μL of primers (0.5 μm of each primer) and 1.5 μL of 1 : 30 diluted cDNA. qRT–PCR data were generated using an Applied Biosystems 7900HT instrument and analysed using SDS software (Applied Biosystems). PCR efficiencies were calculated using the LinReg software. Transcript levels were normalized using the housekeeping gene ACTIN (TC107326). All primer sequences used in this analysis are provided in Table S4.

### Metabolomic analysis of PAs, anthocyanins and flavonoids

Detection of the presence of PAs in hairy roots was done using the DMACA‐HCl protocol (Zhao and Dixon, 2009). Hairy roots (about 150 mg fresh weight) expressing *MtPAR* or *GUS* (as control) were used for PA/anthocyanin quantification. Tissues were ground into powder in liquid nitrogen and extracted three times with 300 μL of methanol containing 0.1% HCl by sonication for 40 min each time. Pooled samples were further extracted with an equal volume of chloroform; the aqueous portion was used for spectrophotometric analysis of anthocyanin at 530 nm with cyanidin 3‐*O*‐glucoside as standard. Epicatechin was used as standard for soluble PA quantification, and the PA dimmer procyanidin B1 was used as standard for insoluble PAs. Reverse‐phase HPLC was used for analysis of cyanidin products by 1‐butanol‐HCl hydrolysis of insoluble PAs and normal phase HPLC coupled to postcolumn DMACA‐derivatization was used for analysis of soluble PAs as described previously (Zhao and Dixon, 2009). PA concentration in alfalfa shoots was measured using DMACA staining and LC‐MS method. Identification of PA monomers and oligomers from *MtPAR* transgenic alfalfa hairy roots was conducted using ESI‐LC‐MS/MS as described previously (Verdier *et al*., 2012; Zhao *et al*., 2011).

For measurement of flavonoid concentration, metabolites were extracted from ~150 mg of fresh hairy root with 2 mL of 80% methanol (containing 18 μg/mL of umbelliferone as internal standard) for 2 h at room temperature. After centrifugation, the supernatants were analysed using a Waters Acquity UPLC system coupled with a quadrupole time‐of‐flight (Q‐TOF) Premier mass spectrometer as described in Zhao *et al*. (2011). Masses of eluted compounds were detected in the negative ESI mode. Metabolites were identified based on mass and retention time relative to authentic standards. Relative abundances were calculated using MET‐IDEA and peaks were normalized by dividing each peak area by the value of the internal standard peak area.

### Yeast hybrid assays

Yeast one‐hybridization assays were performed using Matchmaker^®^ Gold Yeast One‐Hybrid System (Clontech). The promoter of *GmIFS2* (1 to −1634 bp) and *MtIFS1* (1–1525 bp) was PCR amplified from soybean and *M. truncatula* genomic DNA using their gene specific primers for *GmIFS2* and *MtIFS1*. These *IFS* promoters‐driven reporter strains were generated by following the manufacturer's instruction. To construct the transcription factor expressing cassettes, the ORF coding sequences of *MtPAR*,* MtTT8* and *MtWD40‐1* were cloned into *pGBKT7* and *pGADT7*. The resulted vectors were used to transform Y187 yeast strain (Y1H) or Y2H Gold yeast strain (Y2H). The transformed reporter strains were grown on SD/‐Trp‐Leu‐His media containing 20‐30 μm 3‐amino‐1, 2, 4‐triazole (3‐AT) for 3 days at 30 °C. Soybean orthologs of *TT8* and *TTG1* were identified as *GmTT8a* (Glyma10G03140.2), *GmTT8b* (Glyma02G16670.2), *GmWD40a* (Glyma16G04930) and GmWD40b (Glyma06G14180) using blast algorithm. These genes were cloned and ligated into *pGADT7* or *pGBKT7* vectors in reading frame with GL4 activator domain (AD) or DNA binding domain (BD), respectively, and were used for yeast two‐hybrid assays.

### Subcellular localization of MtPAR

MtPAR cDNA were amplified with primers and subcloned into pSPYNE vector (MtPAR‐GFP) (Supplemental Table S4). The resulting constructs were transformed into *Agrobacterium tumefaciens* EHA105 strains. Three‐week‐old *Nicotiana benthamiana* leaves were infiltrated with the mixed bacteria solutions as described previously (Li *et al*., 2015). GFP fluorescence was examined with an Olympus BX53 fluorescence microscope with a 488‐nm excitation and a 505–530 nm filter to record GFP/YFP images and 650–700 nm to record chloroplast autofluorescence.

### Anthocyanidin reductase promoter activation assays

Promoter trans‐activation assays were essentially conducted according to the method described previously (Li *et al*., 2015). A 2‐kb genomic sequence upstream of the translational start codon of *ANR* gene was amplified from *M. truncatula* genomic DNA using the primers (Table S1) and subcloned into p2GW7, which resulted in promoter::*luciferase* reporter constructs. The effector genes (*MtTT8, MtPAR and MtWD40‐1*) were cloned into p2GW7 by LR reaction to form the *35S*::effector constructs using the previously constructed vectors (Wang *et al*., 2011). Transient co‐expression of reporter and effector genes in Arabidopsis leaves were conducted by particle bombardment of Arabidopsis leaves with construct DNAs, according to the method described previously (Ohta *et al*., 2001). The luciferase activities were assayed according to the manufacturer's instructions with a microplate luminometer (Li *et al*., 2015).

## Conflict of Interest

The authors declare no conflict of interest.

## Supporting information


**Figure S1** Phylogenetic analysis of MtPAR and related MYB transcription factors
**Figure S2** Anthocyanin chromatograms in different transgenic *Medicago truncatula* hairy root lines over‐expressing *MtPAR* and *GUS* (control).
**Figure S3** Flavonoid chromatograms in different transgenic *Medicago truncatula* hairy root lines overexpressing *MtPAR* and *GUS* (control).
**Figure S4** Anthocyanin chromatograms in transgenic alfalfa hairy root lines overexpressing *MtPAR*,* MtLAP1*, and *MtPAR‐MtLAP1.*
**Figure S5** Flavonoid chromatograms in different transgenic alfalfa hairy root lines over‐expressing *MtPAR*,* MtLAP1*, and *MtPAR‐MtLAP1*.
**Figure S6** PA production in alfalfa hairy roots over‐expressing *MtPAR, MtLAP1,* and *MtPAR‐MtLAP1*
**Figure S7** Heat map of flavonoid profiles in alfalfa hairy roots over‐expressing *MtLAP1, MtPAR,* and *MtLAP1‐MtPAR*.
**Figure S8** Expression profiles of (iso)flavonoid and PA biosynthetic genes in alfalfa transgenic hairy roots (a) and plants (b) over‐expressing *MtPAR*,* MtLAP1*, and *MtPAR‐MtLAP1*. (c) Alfalfa plants over‐expressing MtPAR and MtPAR‐MtLAP1.
**Figure S9** HPLC analysis of proanthocyanidins.
**Table S1** MYB transcription factor binding sites in the promoter regions of *Isoflavone synthase* (*IFS*) genes.
**Table S2** Expression of genes involved in anthocyanin modification and downstream of BMW regulatory complex in *M. truncatula*
**Table S3** Expression of genes involved in isoflavone biosynthesis in *M. truncatula*.
**Table S4** Primers used in the study.
