# Supplementary material for: Metabolic engineering of proanthocyanidin production by repressing the isoflavone pathways and redirecting anthocyanidin precursor flux in legume
Source: Plant Biotechnol J. 2016 Jan 24;14(7):1604–18. doi: 10.1111/pbi.12524 (PMC5066740; doi:10.1111/pbi.12524)
Supplement: Supplementary file 1 — Figure S1 Phylogenetic analysis of MtPAR and related MYB transcription factors Figure S2 Anthocyanin chromatograms in different transgenic Medicago truncatula hairy root lines over‐expressing MtPAR and GUS (control). Figure S3 Flavonoid chromatograms in different transgenic Medicago truncatula hairy root lines overexpressing MtPAR and GUS (control). Figure S4 Anthocyanin chromatograms in transgenic alfalfa hairy root lines overexpressing MtPAR, MtLAP1, and MtPAR‐MtLAP1. Figure S5 Flavonoid chromatograms in different transgenic alfalfa hairy root lines over‐expressing MtPAR, MtLAP1, and MtPAR‐MtLAP1. Figure S6 PA production in alfalfa hairy roots over‐expressing MtPAR, MtLAP1, and MtPAR‐MtLAP1 Figure S7 Heat map of flavonoid profiles in alfalfa hairy roots over‐expressing MtLAP1, MtPAR, and MtLAP1‐MtPAR. Figure S8 Expression profiles of (iso)flavonoid and PA biosynthetic genes in alfalfa transgenic hairy roots (a) and plants (b) over‐expressing MtPAR, MtLAP1, and MtPAR‐MtLAP1. (c) Alfalfa plants over‐expressing MtPAR and MtPAR‐MtLAP1. Figure S9 HPLC analysis of proanthocyanidins. Table S1 MYB transcription factor binding sites in the promoter regions of Isoflavone synthase (IFS) genes. Table S2 Expression of genes involved in anthocyanin modification and downstream of BMW regulatory complex in M. truncatula Table S3 Expression of genes involved in isoflavone biosynthesis in M. truncatula. Table S4 Primers used in the study. [file PBI-14-1604-s001.doc]

**MtPAR represses isoflavone biosynthesis and directs anthocyanidin precursors into the proanthocyanidin pathway in legumes**

**Penghui Li^a^, Shujun Ge^b,c^, Qiang Dong^a^, Xianzhi He^c,d^, Jerome Verdier^e^, Jian Zhao^a^****

**Online Supporting Figures, Tables, and Data:**

**Supporting Figure S1**. Phylogenetic analysis of MtPAR and related Myb transcription factors. Phylogenetic tree of MYB transcription factors was constructed by the neighbor-joining method with 1,000 bootstrap replicates by the software MEGA version 5.0 (<http://mega.software.informer.com/5.0/>). The Myb transcription factors from different plants and their GenBank accession number: Grapevine ([Vitis vinifera](http://en.wikipedia.org/wiki/Vitis_vinifera)) Mybs VvMybPA1 (CAJ90831), VvMyb5a (NP_001268108.1), VvMyb5b (NP_001267854.1), and VvMybPA2 (EU919682); *Arabidopsis thaliana* Mybs AtPAP1 (NP_176057), AtMyb3 (Q9S9K9.1), AtMyb4 (Q9SZP1.1), AtMybL2 (AEE35154.1), AtMyb5 (Q38850.1), and AtTT2 (NP_198405.1); *Petunia x hybrida* Myb PH4 (AAY51377.1); persimmon (*Diospyros virginiana*) DkMyb4 (BAI49721.1), *Lotus japonica* LjTT2a (BAG12893.1), white clover (*Trifolium repens*) Myb TaMyb14 (AFJ53054.1); *Medicago truncatula* MtMyb14 (KEH32454.1), MtPAR (XP_003627264.1), MtLAP1 (ACN79541), MtMyb3(XP_003616388.1), MtMyb5 (XP_003601609.1); PopularPtMyb134 (ACR83705.1), MtLAP1 (ACN79541), MtMyb3(XP_003616388.1), MtMyb5 (XP_003601609.1).

**Supporting Figure S2.** Anthocyanin chromatographs in different transgenic *Medicago truncatula* hairy root lines over-expressing *MtPAR* and *GUS* (control). Photos showed representative colors of different hairy roots.


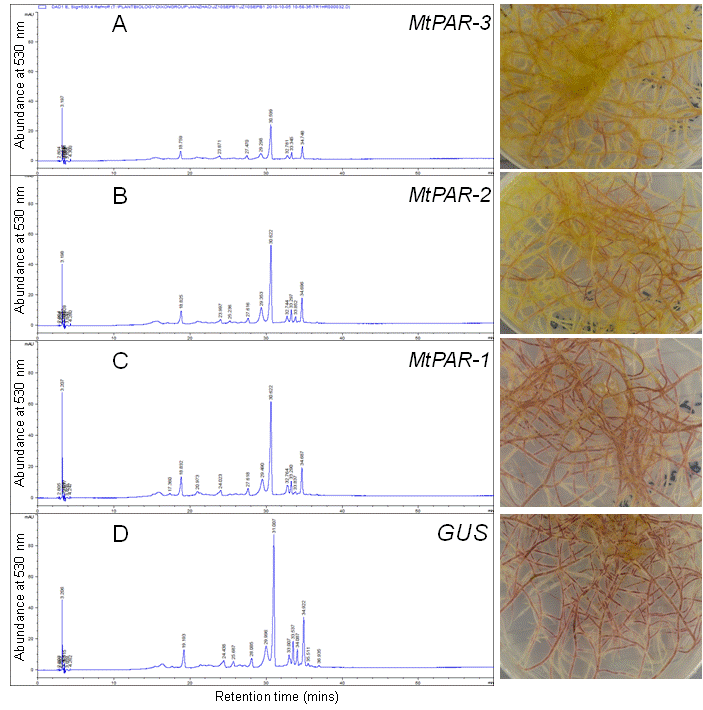


**Supporting Figure S3.** Flavonoid chromatograms in different transgenic *Medicago truncatula* hairy root lines over-expressing *MtPAR* and *GUS* (control). HPLC traces are representatives from at least three independent experiments.


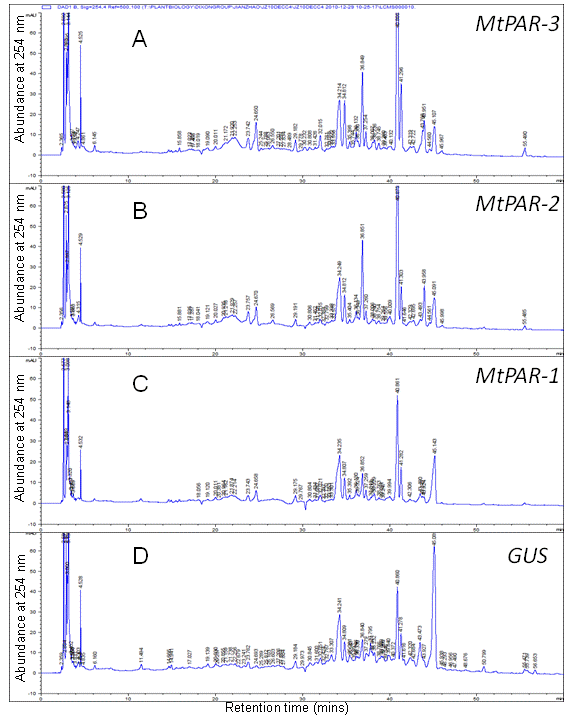


**Supporting Figure S4.** Anthocyanin chromatograms in different transgenic alfalfa hairy root lines over-expressing *MtPAR, MtLAP1*, and *MtPAR-MtLAP1*. Photos showed representative colors of different hairy roots. HPLC traces are representatives from at least three independent experiments.

**
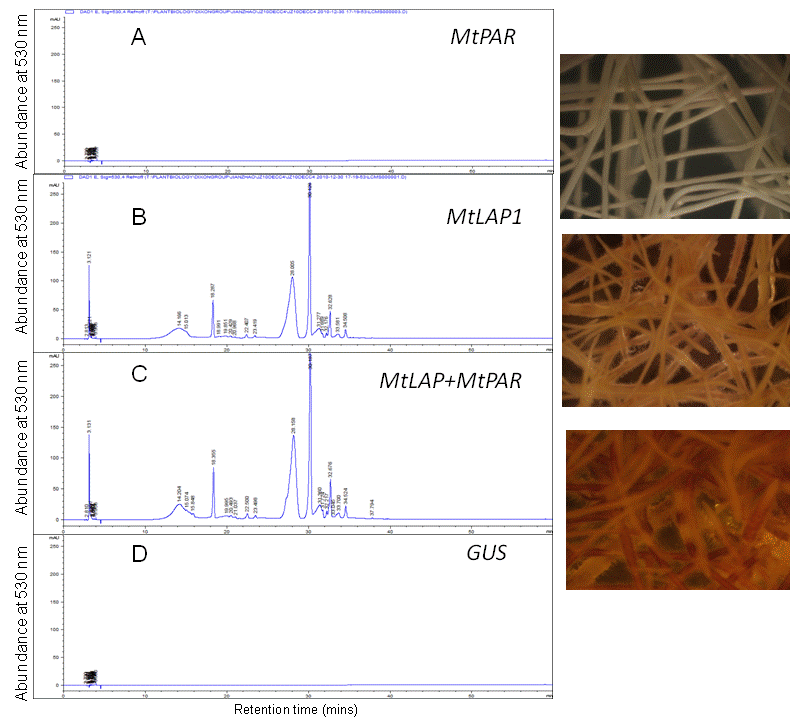
**

**Supporting Figure S5.** Flavonoid chromatograms in different transgenic alfalfa hairy root lines over-expressing *MtPAR*, *MtLAP1*, and *MtPAR*-*MtLAP1*. HPLC traces are representatives from at least three independent experiments.

**
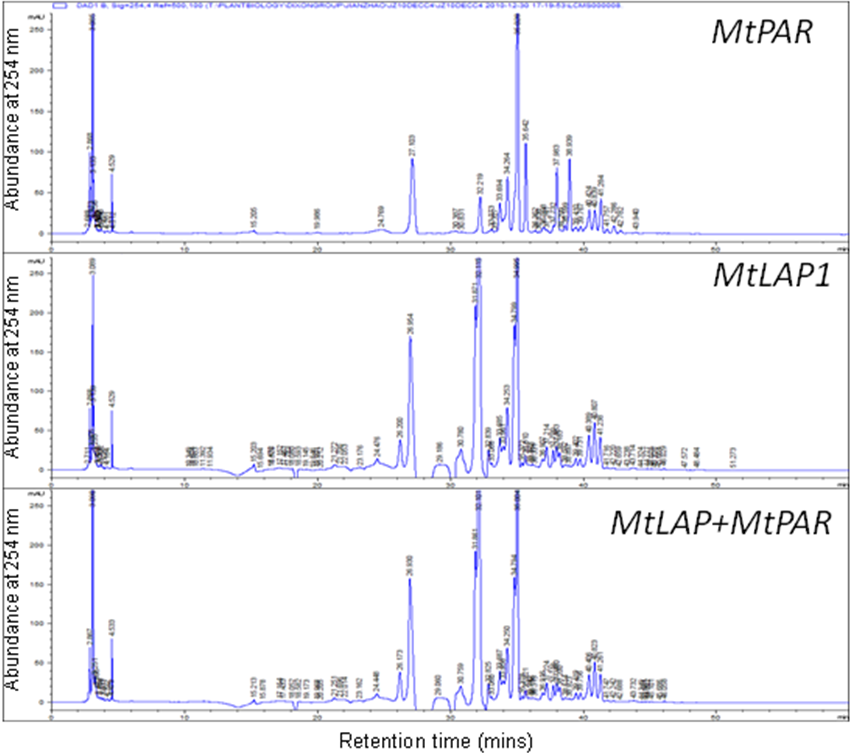
**

**Supporting Figure S6.** PA production in alfalfa hairy roots over-expressing *MtPAR, MtLAP1, MtPAR-MtLAP1*.

**A.** DMACA staining of alfalfa hairy roots over-expressing *MtPAR*, *MtLAP1*, *MtPAR-MtLAP1* and *GUS* for PA detection. **B.** *MtPAR-MtLAP1* co-expression in hairy roots under light imaging (left panel), GFP imaging of hairy root monitoring expression of *MtPAR* (middle panel), and light imaging after DMACA staining of hairy roots (right panel). Photos showed representative colors of different hairy roots.

**Supporting Figure S7.** Heatmap of flavonoid profiles in alfalfa hairy roots over-expressing *MtLAP1*, *MtPAR*, *MtLAP1-MtPAR* and *GUS* (CK). Values represent averages from at least three biological replicates.


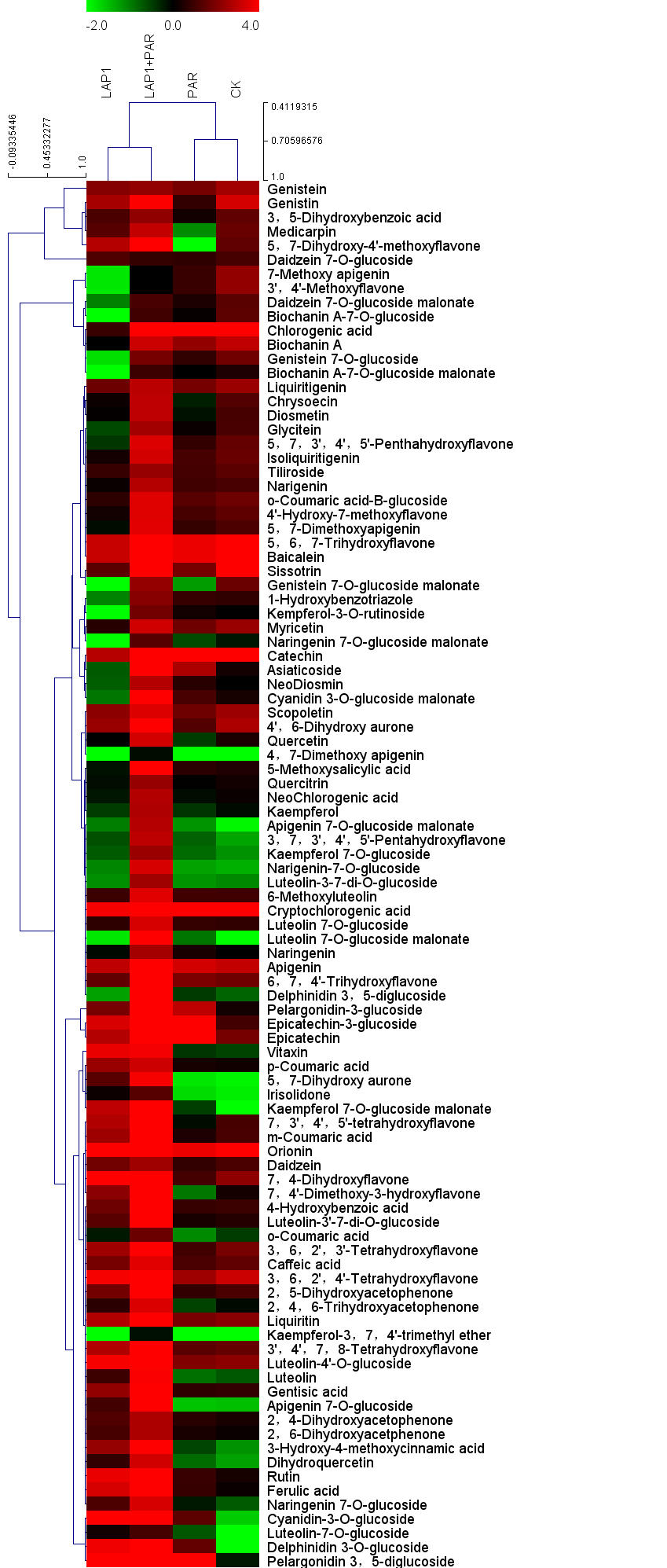


**Supporting Figure S8.** Expression profiles of (iso)flavonoid and PA biosynthetic genes in alfalfa transgenic hairy roots (**A**) and plants (**B**) over-expressing *MtPAR*, *MtLAP1*, and *MtPAR*-*MtLAP1*. Data are expressed as ratio to GUS control in average with standard deviations from three biological replicates. **(C**) Alfalfa plants over-expressing *MtPAR* and *MtPAR*-*MtLAP1*.

**
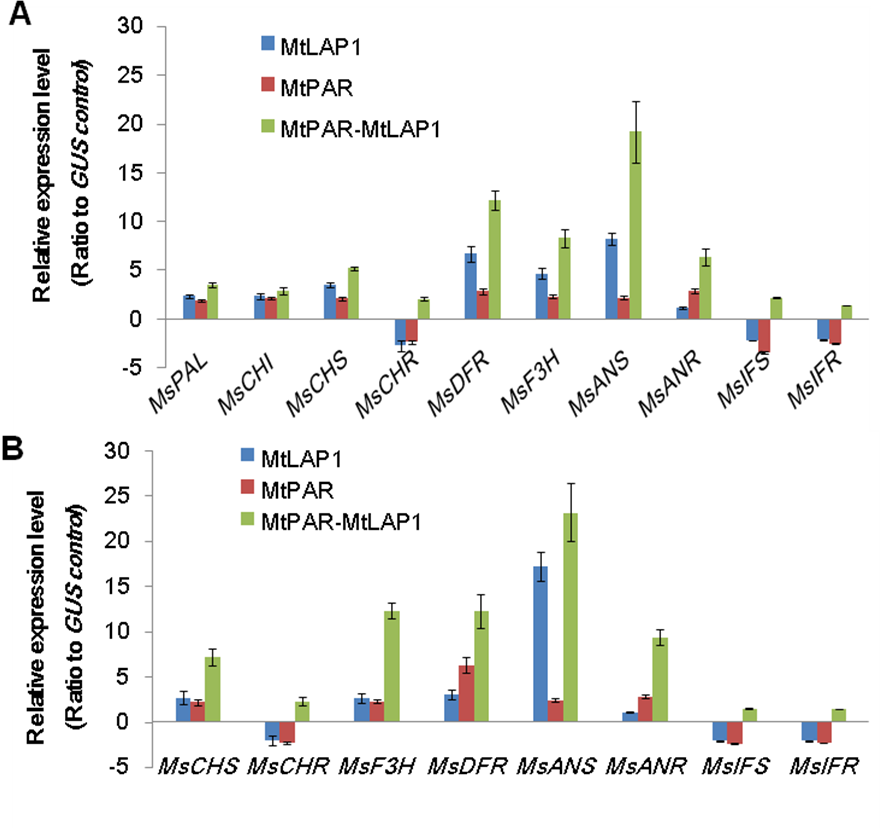
**

**Supporting Figure S9.** HPLC analysis of proanthocyanidins. Left panels: standards, pelargonidin, cyanidin, and delphinidin; Right panels: chromatograms for *MtPAR*, *MtPAR*-*MtLAP1* and *GUS* transgenic alfalfa plants. **B.** Identification of epicatechin, epicatechin glucoside, and PA oligomers in soluble PA extracts from *MtPAR*-*MtLAP1* transgenic alfalfa with ESI-LC-MS/MS. These compounds were not detected in control plants. Left panels (from Top to Bottom): epicatechin ion peak MS1, (m/z 288); MS2, ion peak (m/z 244) from breakdown of epicatechin; Right panels (from Top to Bottom): epicatechin dimer (B1) ion peak (m/z 577), MS2 ion peak (m/z 288.5) from breakdown of epicatechin dimer; epicatechin glucoside ion peak (m/z 450.5) was also shown in arrows.


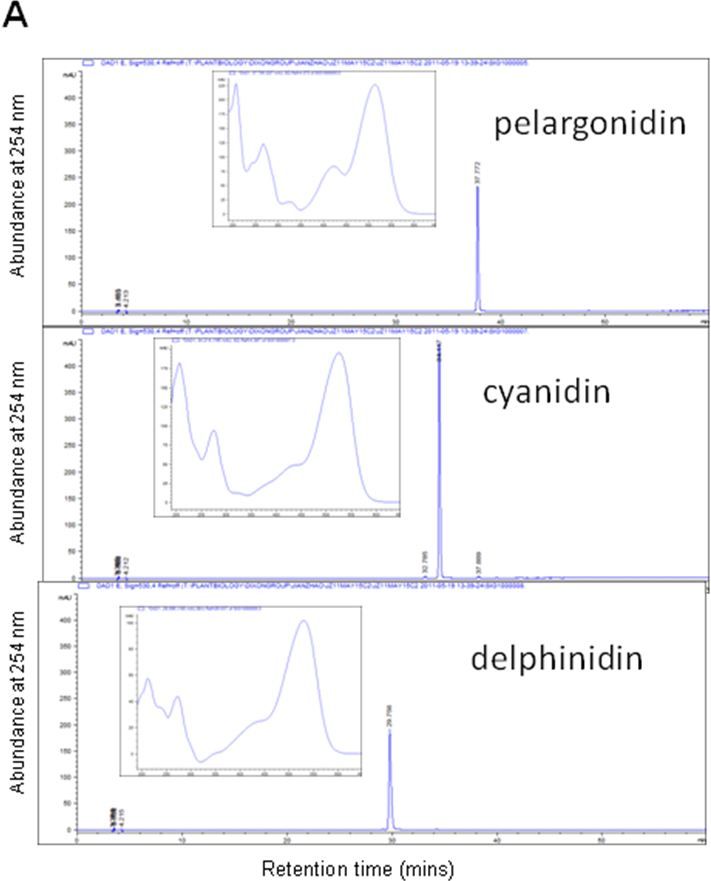

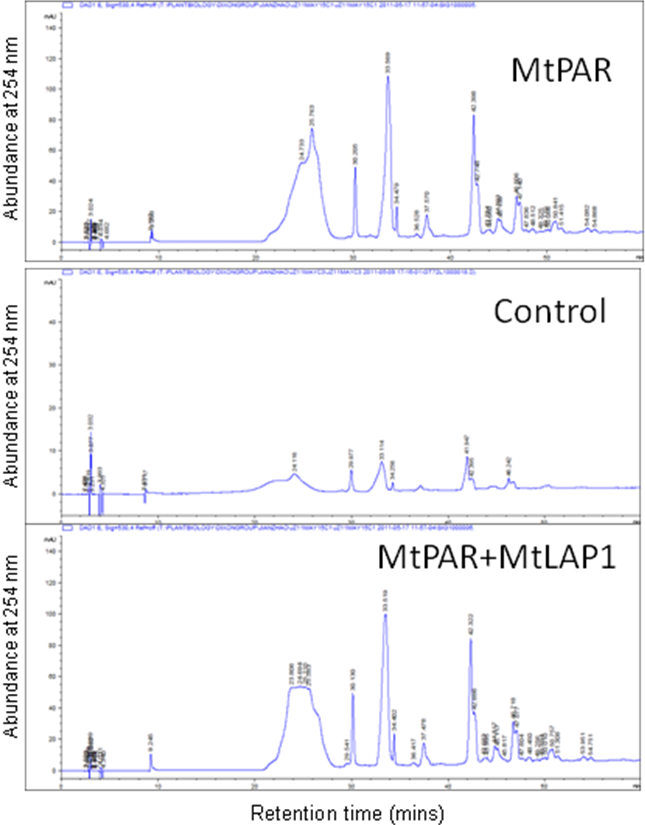


**
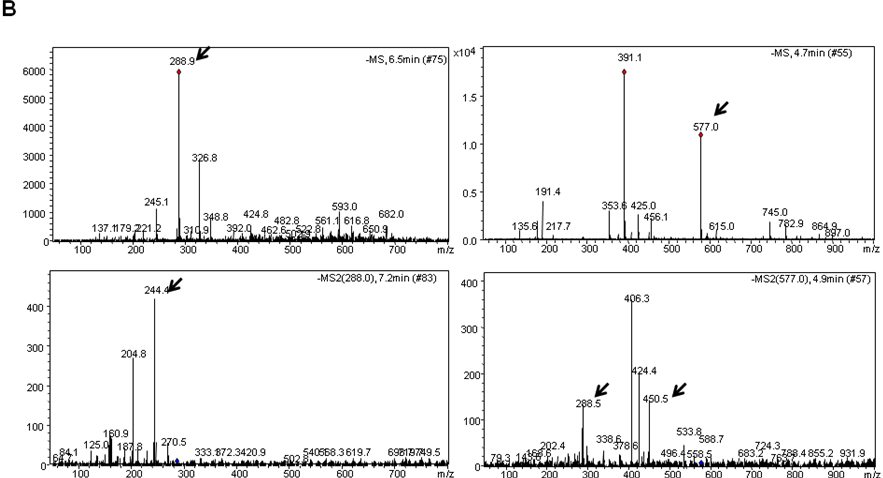
**

**Supporting Table S1.** Expression of genes involved in the anthocyanin modification and putative genes downstream of BMW regulatory complex in *M. truncatula*.

| Probeset | Gene description | IMGAG gene ID (V4.0) and Name | MtPAR/GUS  microarray | MtPAR/GUS  qRT-PCR |
| --- | --- | --- | --- | --- |
| Mtr.44986.1.S1_at | TC98548 /FEA=mRNA /DEF=similar to UP|Q9FID1 (Q9FID1) Acyltransferase-like protein (At5g39090), partial (13%) | Medtr6g015830.1.  MtMATE2 | 0.68 | 0.79 |
| Mtr.37182.1.S1_at | TC100057 /FEA=mRNA /DEF=similar to GB|BAB02518.1|11994477|AB028618 anthocyanin 5-aromatic acyltransferase/benzoyltransferase-like protein {Arabidopsis thaliana;} , partial (17%) | Medtr6g015815.1,  MtMATE2 | 1.05 | 0.87 |
| Mtr.39747.1.S1_at | TC105632 /FEA=mRNA /DEF=weakly similar to UP|Q9AR45 UDP-glucose:flavonoid 3-O-glucosyltransferase, partial (35%) | Medtr4g128690.1, MtUGT78G1 | 0.85 | 1.03 |
| Mtr.19945.1.S1_at | IMGAG|1095.m00006 /FEA=mRNA /DEF=anthocyanin acyltransferase AC146566.7.61 22887 21481 mth2-71p5 | Medtr3g028210.1,  MtMaT5 | 1.28 | 1.02 |
| Mtr.15281.1.S1_at | IMGAG|781.m00001 /FEA=mRNA /DEF=Transferase AC125389.32.1 1208 2641 mth2-12a18 01/13/05 | Medtr3g087640.1,  MtMaT6 | 0.86 | 0.83 |
| Mtr.45072.1.S1_at | TC98718 /FEA=mRNA /DEF=weakly similar to UP|LGT_CITUN (Q9MB73) Limonoid UDP-glucosyltransferase (Limonoid glucosyltransferase) (Limonoid GTase), partial (32%) | Medtr1g107365.1,  MtUGT | 1.11 | 0.91 |
| Mtr.51063.1.S1_at | IMGAG|729.m00016 /FEA=mRNA /DEF=Multi antimicrobial extrusion protein MatE AC121237.19.151 69977 73532 mth2-22g11 | Medtr1g100180.1,  Mt MATE2 | 0.83 | 0.68 |
| Mtr.47151.1.S1_at | 1695.m00045 /FEA=mRNA /DEF=AC147472.15 118222 120283 mth2-24n18 weakly similar to UP|O24261 (O24261) Glutathione S-transferase | Medtr3g064700.1  MtGST | 0.82 | 0.78 |
| Mtr.38588.1.S1_at | TC103118 /FEA=mRNA /DEF=similar to UP|Q675M4 (Q675M4) Myc-like anthocyanin regulatory protein, partial (45%) | Medtr8g098275.1,  MtEGL3 | 0.92 | 1.92 |
| Mtr.35850.1.S1_at | TC97762 / /DEF=similar to GB|AAM61951.1|21541739|AF516172 transcription factor WRKY44 {Arabidopsis thaliana;} , partial (11%) | Medtr7g100510.1,  MtTTG2 | 1.78 | 2.34 |
| Mtr.22479.1.S1_at | 1606.m00039 /FEA=mRNA /DEF=AC135317.10 9174 16609 mth2-10p4 weakly similar to UP|Q9FEA1 Anthocyanin 1 | Medtr1g072320.1,  MtTT8 | 1.55 | 4.54 |
| Mtr.28737.1.S1_at | BI312112 /FEA=mRNA /DEF=similar to UP|Q8LJS8 (Q8LJS8) Homeodomain protein GhHOX1, partial (27%) | Medtr2g101720.1,  MtGL2 | 2.22 | 5.31 |

**Supporting Table S2.** Expression of genes involved in isoflavone biosynthesis in *M. truncatula*.

| Probeset | Gene Description | IMGAG gene ID (V4.0) and Name | MtPAR/GUS  microarray | MtPAR/GUS  qRT-PCR |
| --- | --- | --- | --- | --- |
| Mtr.37751.1.S1_at | TC101337 /FEA=mRNA /DEF=similar to UP|7MT9_MEDSA (O22309) Isoflavone-7-O-methytransferase 9 (Isoflavone-O-methytransferase 9) (7IOMT-9), partial (37%) | Medtr4g090840.1,  IOMT | 0.28 | 0.02 |
| Mtr.40504.1.S1_at | TC107427 /FEA=mRNA /DEF=weakly similar to UP|Q6YI95 O-methyltransferase, partial (79%) | Medtr6g091680.1,  IOMT | 0.37 | 0.12 |
| Mtr.13532.1.S1_at | TC98300 /FEA=mRNA /DEF=similar to UP|Q9XFX1 (Q9XFX1) Cytochrome P450, partial (46%) | Medtr5g016440.1,  P450 | 0.30 | 0.16 |
| Mtr.43713.1.S1_at | TC95902 /FEA=mRNA /DEF=similar to UP|O80407 (O80407) Chalcone synthase, partial (95%) | Medtr3g088675.1,  CHS | 0.13 | 0.01 |
| Mtr.5125.1.S1_s_at | AW688509 /FEA=mRNA /DEF=weakly similar to UP|O65679 (O65679) Isoflavone reductase-like protein, partial (38%) | Medtr4g070360.1,  IFS | 1.02 | 0.67 |
| Mtr.27878.1.S1_at | BF006345 /FEA=mRNA /DEF=weakly similar to UP|O49820 (O49820) Isoflavone reductase-like protein, partial (61%) | Medtr3g007970.1,  IFR | 0.99 | 0.52 |
| Mtr.40277.1.S1_x_at | TC106940 /FEA=mRNA /DEF=homologue to UP|Q84QI4 (Q84QI4) Isoflavone synthase, partial (28%) | Medtr4g088170.1,  IFS | 0.98 | 0.62 |
| Mtr.25013.1.S1_at | 1785.m00044 /FEA=mRNA /DEF=AC155890.1 38120 35370 mth2-49p3 similar to UP|Q9SDZ2 (Q9SDZ2) 2'-hydroxy isoflavone/dihydroflavonol reductase homolog (Fragment) | Medtr7g074820.1,  I2'H | 0.97 | 0.79 |
| Mtr.29301.1.S1_at | CB893405 /FEA=mRNA /DEF=weakly similar to UP|7MT6_MEDSA (O22308) Isoflavone-7-O-methytransferase 6 (Isoflavone-O-methytransferase 6) (7-IOMT-6) , partial (22%) | Medtr7g034960.1,  I7OMT | 0.97 | 0.46 |
| Mtr.43009.1.S1_at | TC94324 /FEA=mRNA /DEF=UP|Q6WNR0 (Q6WNR0) Isoflavone 2'-hydroxylase, complete | Medtr4g094775.1,  I2'H | 0.96 | 0.86 |
| Mtr.43916.1.S1_at | TC96312 /FEA=mRNA /DEF=UP|Q9LKI6 (Q9LKI6) Isoflavone reductase, partial (35%) | Medtr5g020800.1,  IFR | 0.94 | 0.44 |

**Supporting Table S3.** MYB transcription factor binding sites identified from promoter regions of isoflavone synthase (IFS) genes from different legume plants (*Mt, Medicago truncatula; Gm, Glycine max; Pv, Phaseolus vulgaris*).

**Supporting Table S4.**  Primers used in the study

**Supporting Table S3.** MYB transcription factor binding sites identified from promoter regions of isoflavone synthase (IFS) genes

from different legume plants (*Mt, Medicago truncatula; Gm, Glycine max; Pv, Phaseolus vulgaris*).

| Cis-elements | Sequence  and AC site | Number of binding sites | | | | |
| --- | --- | --- | --- | --- | --- | --- |
|  |  | *MtIFS1*  (Medtr7g027960) | MtIFS2  (Medtr7g028020) | GmIFS1  (Glyma.07G202300) | GmIFS2  (Glyma.13G173500) | PvIFS  (Phvul.003G051700) |
| MYB1AT | WAACCA,  S000408 | 2 | 3 | 3 | 4 | 1 |
| MYB1LEPR | GTTAGTT,  S000443 | 2 | 0 | 1 | 0 | 0 |
| MYB2AT | TAACTG,  S000177 | 2 | 1 | 0 | 1 | 0 |
| MYB2CONSENSUSAT | YAACKG,  S000409 | 5 | 2 | 3 | 1 | 1 |
| MYBCORE | CNGTTR,  S000176 | 7 | 5 | 7 | 3 | 2 |
| MYBCOREATCYCB1 | AACGG,  S000502 | 0 | 1 | 2 | 0 | 1 |
| MYBGAHV | TAACAAA,  S000181 | 0 | 0 | 0 | 2 | 1 |
| MYBPLANT | MACCWAMC,  S000167 | 2 | 0 | 0 | 1 | 0 |
| MYBPZM | CCWACC,  S000179 | 2 | 1 | 0 | 2 | 1 |
| MYBST1 | GGATA,  S000180 | 2 | 5 | 5 | 1 | 4 |

Note: About 1.5 Kb promoter regions were used to search the *cis*-elements known as MYB binding sites using PLACE algorithm.

**Supporting Table S4. Primers used in the study**

| **Oligo names** | **Sequences(5'->3')** | **Notes** |
| --- | --- | --- |
| MtPALqpcrF | TTGCCAAAAGAGGTTGAAAGTG | MtPAL qPCR  MtPAL qPCR |
| MtPALqpcrR | TGTTTGGAATTGTTGGGTTTCC |
| MtC4HqpcrF | ATCCCACTCCTCGTACCACA | MtC4H qPCR  Medtr5g075450.1 |
| MtC4HqpcrR | CAAGCCACCATGCATTGACC |
| MtCHSqpcrF | CCACGACACCATCCTAAATTGTATC | MtCHS qPCR  Medtr5g007713.1 |
| MtCHSqpcrR | TGGTGTGACTAATGCCTTTTTGAC |
| MtCHI1qpcrF | CACGCTGTTTCCCCTGATCT | MtCHI1 qPCR  Medtr1g115840.1 |
| MtCHI1qpcrR | TCAACAACGCCGGTAATCTTG |
| MtCHI2qpcrF | AGAGATTAAGGGTGCACAGTATGGT | MtCHI 2qPCR  Medtr1g115820.2 |
| MtCHI2qpcrR | TCATCGGCTGCCAAACG |
| MtCHI3qpcrF | TTTCCGGCAGTGGTGACA | MtCHI3 qPCR  Medtr3g093980 |
| MtCHI3qpcrR | TGCACCACCGAGGAAATAGG |
| MtF3HqpcrF2 | AATTACTACCCAAAATGCCCTCAA | MtF3H qPCR  Medtr8g075890.1 |
| MtF3HqpcrR2 | GCCAGGGTCAGTGTGTCGTT |
| MtF3′5′HqpcrF | AGACATGGTTTTCGCGGACT | MtF3,5,H qPCR |
| MtF3′5 ′HqpcrR | CCCATTTCGTCCCCTCGAAT |
| MtIFR2qpcrF | AAGCAAGTGGACATCGTAATCTCA | MtIFR2 qPCR  Medtr4g070340.2 |
| MtIFR2qpcrR | GCAGCAAGGATCTTATATTGATCAGA |
| MtIFR1qpcrF | AAAAGCGAGTATCCGAAGAGTAATTG | MtIFR1 qPCR  Medtr5g020760.1 |
| MtIFR1qpcrR | GCGTGGCAACAAAGGTAAGTG |
| MtIFSqpcrF1 | CAATCCTCCGAGTCCCAAAC | MtIFS qPCR  Medtr1g022445.1 |
| MtIFSqpcrR1 | GGGTTATCCAAAAGGTGAAGATGA |
| MtDFR1qpcrF1 | TCGTCCACTTGGATGATCTTTG | MtDFR1 qPCR  Medtr1g022445.1 |
| MtDFR1qpcrR1 | CTCCCTTCTACTTCCATATGCTCAA |
| MtANSqpcrF1 | TCCACCTCGCACTTTTGCT | MtANS qPCR  Medtr5g011250.1 |
| MtANSqpcrR1 | TCTTCTCCTCCTCATCCTTCCTAA |
| MtANRqpcrF1 | TTGTGGCAGAGAAAGAATCAACTT | MtANR qPCR  Medtr3g083910.1 |
| MtANRqpcrR1 | CTCGGGAACACTGGTATTGTGA |
| MtLARRTF | GGAATAGCCGCAGAAAATTG | MtLAR qPCR |
| MtLARRTR | CATGGCAACAAAGCTCTCAA |
| MtWD40-1qpcrF1 | ATTCCCCACAACAACGTATCG | MtWD40-1 qPCR |
| MtWD40-1qpcrR1 | ATGCGGTTTGTGTATTCTTCGA |
| MtTT8qpcrF1 | GCCAGCAACTAAGAGAACTCTATGAA | MtTT8 qPCR |
| MtTT8qpcrR1 | GGACGACGAGTTGGTGGATT |
| MtWD40YF | AGAATTCATGGATAATTCAACAC | MtWD40-1 cloning and yeast hybrid |
| MtWD40YR | AGGATCCTCAAACCCTCAAAAGC |
| MtTT8YF | AGAATTCATGGCTGCTCCATCTCCA | MtTT8 cloning and  yeast hybrid |
| MtTT8YR | AGGATCCCTAAATGTTGTGAGGTA |
| MtPARYF | AGAATTCATGGTTAGAAGTCCTAAG | MtPAR cloning and yeast hybrid |
| MtPARYR | AGGATCCTCAATCATTTTCAAGTCC |
| Mt4CLqpcrF | TTGTTCCGCCGTTGGTTTTG | Mt4CL qPCR  Medtr5g007720.1 |
| Mt4CLqpcrR | CTCCTTCCCTAACGGTGCA |
| MtF3'HqpcrF1 | CACCAGCTCAAACTCTCACCTATC | MtF3'H qPCR  Medtr4g109470.1 |
| MtF3'HqpcrR1 | TTCGCGAACAAAACTGGATTC |
| MtFLSqpcrF2 | CACATGAGGTCGTGGTTGGA | MtFLS qPCR |
| MtFLSqpcrR2 | TTTGGCAGGGTTTTGATCATT |
| UGT83F1qpcrF1 | GTTTTGGCATTCCCATTTGG | UGT83F1 qPCR |
| UGT83F1qpcrR1 | GAGCCTCTGTAGCAATTTTTTTCAC |
| UGT72L1qpcrF1 | GGAGAATCCACATGATTCTTTT | UGT72L1 qPCR |
| UGT72L1qpcrR1 | TCTGAATGAGAATTCAGGGAAA |
| MtMATE1qpcrF | CAGAGAGCATAGCCGTGCAA | MtMATE1 qPCR |
| MtMATE1qpcrR | TTCTGCGCTTGGAGAAACCT |
| MtPARqpcrF1 | TTGCCATCACCATCTTCTGTTT | MtPAR qPCR  First pair of primers |
| MtPARqpcrR1 | GGTGCTACATGTCCACATTTGC |
| Mt PARqpcrF2 | TCGACACAATCATGGCAAATG | MtPAR qPCR  Second pair of primers |
| Mt PARqpcrR2 | CCTTCAGTCTCCTAGGCTTTGG |
| MtLAP1qpcrF | GCGCATGGACTTACAAGGA | MtLAP1 qPCR |
| MtLAP1qpcrR | CTCTGTTGATGTTGGGGCT |
| MtMATE2qpcrF | CAGCTGTTATGCTTTGCCTTGA | MtMATE2 qPCR |
| MtMATE2qpcrR | CAAGGTGGCCAGCCAGAA |
| Actin2qpcrF | ACTCACACCGTCACCAGAATCC | Actin2 qPCR |
| Actin2qpcrR | TCAATGTGCCTGCCATGTATGT |
| MtUbiquitinR | AACTCTTGGGCAGGCAATAA | Ubiquitin qPCR |
| MtUbiquitinF | GCAGATAGACACGCTGGGA |
| *MtIFS1Fpro* | AGAGCTCAGCAATTACAACCCATC | Reporter construct |
| *MtIFS1Rpro* | AACTAGTGATATGTTGCTATAGTA |
| *GmIFS2pro-1634F* | GACAGAATTCCACATTCCATAAACTCT | Reporter construct |
| *GmIFS2pro-1097F* | GACAGAATTCGCAAAGAGAACCAAAACA |
| GmIFS2-654F | GACAGAATTCCCAATCTCATAATGAATCTA |
| *GmIFS2pro-255F* | GACAGAATTCCATTTGTCACTCTTAATATA |
| GmIFS2-1R | GACAACGCGTGGTATGTTGAAGCTAGT |
| MsCHRqRTF | TCTGTCGCCACTGTTCTTCC | qRT-PCR |
| MsCHRqRTR | ATTCCGTGTGCGTTGCAAAA |
| MsCHSqRTF | ACTATTTGGAGATGGAGCTGCTG | qRT-PCR |
| MsCHSqRTR | ACTATTTGGAGATGGAGCTGCTG |
| MsANRqRTF | AACGAACTCGAAGGGACTGG | qRT-PCR |
| MsANRqRTR | GGATAACCCCAAGTGGGTGG |
| MsDFRqRTF | TGCGCGACCCAGATAACATA | qRT-PCR |
| MsDFRqRTR | GGTCAGCCTTCCAAAGAGACA |
| MsIFS2qRTF | CTTCGGCGAATACAGCCTCA | qRT-PCR |
| MsIFS2qRTR | AAAGTGTCGAGGAAGACGCC |
| MsF3H qRTF | TGGCACAGGCAAAAACCATC | qRT-PCR |
| MsF3H qRTR | TTCACCTCGTCGATTCCAGC |
| MsCHIqRTF | CGCAATCACTGTGGAGAACCTT | qRT-PCR |
| MsCHIqRTR | ATGGTCAATCCTCTCTCCCCTG |
| MtGL2qpcrF | ACCCTACGAGAGACCATAAA | Medtr2g101720 qPCR |
| MtGL2qpcrR | CTCTCAGCCTCTCTACCTCA |
| MsANSqRTF | ACCCAATTTGCCCTCAACCA | qRT-PCR |
| MsANSqRTR | AAAGTTGCAAACCTGGCACC |
| MsIFRqRTF | ACGAGGCAGTTGAGCCAGTTAG | qRT-PCR |
| MsIFRqRTR | GCGTGGCAACAAAGGTAAGTGT |
| MsPALqRTF | TGAAGCGTATGGTGGCAGAG | qRT-PCR |
| MsPALqRTR | CCCATTCACTGCTGGCCTTA |
| GmPAL1qpcrF | AGCAACACAACCAGGATGTCAA | qRT-PCR |
| GmPAL1qpcrR | CAATTGCTTGGCAAAGTGCA |
| GmC4HqpcrF | AGGCGAGATCAACGAAGACAAC | qRT-PCR |
| GmC4HqpcrR | GTTCACAAGCTCAGCAATGCC |
| Gm4CLqpcrF | AGGCAATGTACGTGGACAAGCT | qRT-PCR |
| Gm4CLqpcrR | TCCGAGAGGACAGAGAAGTGGA |
| GmCHRqpcrF | CAAAGCCATTGGAGTCAGCAA | qRT-PCR |
| GmCHRqpcrR | CCATGCAAGGTTCATCTCCACT |
| GmCHI1AqpcrF | GGCGCTGAATACTCAAAGAAGG | qRT-PCR |
| GmCHI1AqpcrR | AGAGGCACCAGGTGCAAAATT |
| GmCHI1B1qpcrF | AGCTGAATTGCTCGACTCCCT | qRT-PCR |
| GmCHI1B1qpcrR | CAGATTGCATATGTGCCACACA |
| GmIFS1qpcrF | AGAATTCCGTCCCGAGAGGTT | qRT-PCR |
| GmIFS1qpcrR | TGCCATTCCTGAAGTAGCCAA |
| GmIFS2qpcrF | AATGTGCCCTGGAGTCAATCTG | qRT-PCR |
| GmIFS2qpcrR | GGCGTCACCACCCTTCAATAT |
| GmIFRqpcrF | AGATGGAAATGTGAAAGGAGCG | qRT-PCR |
| GmIFRqpcrR | TGTGCACGGCTTTGTTCAAG |
| GmF3HqpcrF | TTACCTGGCCCAGGAGAAAAC | qRT-PCR |
| GmF3HqpcrR | ATTCCGGCAAGAGAAATCACTG |
| GmDFR1qpcrF | TTGTTGTCGGTCCCTTTCTGA | qRT-PCR |
| GmDFR1qpcrR | GTGGACGAATTGACCTTGCTTT |

**Reference：**

Gutierrez-Gonzalez JJ, Guttikonda SK, Tran LS, Aldrich DL, Zhong R, Yu O, Nguyen HT, Sleper DA.Differential expression of isoflavone biosynthetic genes in soybean during water deficits. 2010 Jun; Plant Cell Physiol. 51(6):936-48.
